# Supplementary material for: Hardware-tailored diagonalization circuits
Source: npj Quantum Inf. 2024 Nov 21;10(1):122. doi: 10.1038/s41534-024-00901-1 (PMC11581980; doi:10.1038/s41534-024-00901-1)
Supplement: Supplementary file 1 — Supplementary Information [file 41534_2024_901_MOESM1_ESM.pdf]

Supplementary Material (SM) to Hardware-Tailored Diagonalization Circuits

Daniel Miller,[1, 2, 3, *] Laurin E. Fischer,[3] Kyano Levi,[1] Eric J. Kuehnke,[1]
Igor O. Sokolov,[3, 4] Panagiotis Kl. Barkoutsos,[3, 4] Jens Eisert,[1] and Ivano Tavernelli[3]

[1] *Dahlem Center for Complex Quantum Systems, Freie Universität Berlin, 14195 Berlin, Germany*
[2] *Institute for Theoretical Nanoelectronics (PGI-2), Forschungszentrum Jülich, 52428 Jülich, Germany*
[3] *IBM Quantum, IBM Research Europe – Zurich, Säumerstrasse 4, 8803 Rüschlikon, Switzerland*
[4] *Current address: PASQAL, 7 rue Léonard de Vinci, 91300 Massy, France*

CONTENTS

I. Practicality of HT readout circuits despite their larger bias 2

II. General Clifford circuits have huge overheads 2

III. Completing a stabilizer group 3

IV. Optimally allocating shots to readout circuits 4

V. Pauli grouping algorithms 4

VI. Preprocessing time vs. runtime savings for a 52-qubit example 6

VII. Exploring hyperparameter choices for molecular Hamiltonians 9
- Choice of molecules 9
- Influence of the value function 10
- Carefully balancing hyperparameters 10
- No gain through updating subgraphs on the fly 11

VIII. Detailed investigation of Pauli groupings for random Hamiltonians 11

IX. Hamiltonian exponentiation 13

X. How to compute intersections of affine subspaces 15

XI. Dependence of HT-identifiability on the cutoff 15

XII. Breaking the exponential runtime for factorizable circuits 16

XIII. Details about the experiment 16
- Choice of Hamiltonian and TPB grouping 16
- GC grouping of the Hamiltonian 17
- HT grouping of the Hamiltonian 17
- Experimental procedure 17
- Correct interpretation of the measurement data 17

XIV. Basis sets beyond STO-3G 18

* daniel.miller@fu-berlin.de

I. PRACTICALITY OF HT READOUT CIRCUITS DESPITE THEIR LARGER BIAS

Here we discuss use cases in which *hardware-tailored* (HT) readout circuits have a practical advantage over conventional *tensor product bases* (TPBs). In Fig. 3 of the main text, we have identified two important parameter regimes: the low-shot (high-error) and the high-shot (low-error) regime. While HT readout circuits outperform TPBs in the low-shot regime, the opposite is true in the high-shot regime. In the high-shot regime, the bias b (noise floor) is larger for HT than for TPB because HT circuits make use of two-qubit gates, which have higher error rates than single-qubit gates, however, our HT circuits have extremely low two-qubit gate counts (here at most 4, see Tab. VII in SM Sec. XIII). Hence, we can attribute the small but clearly visible difference between b_{HT} and b_{TPB} to the fact that we measured a product state whose preparation requires no two-qubit gates at all. For practical applications, on the other hand, one would have to prepare highly entangled quantum states. Whenever the number of two-qubit gates in the preparation circuit outweighs those in the readout circuit, which can always be achieved by restricting the search space of readout circuit templates, the difference between b_{HT} and b_{TPB} will be negligible. Here it is important to point out that we can flexibly trade off the readout circuit complexity and the obtainable shot reduction over TPBs. For example, we can impose a gate-count limit when constructing HT readout circuits.

Having said this, HT readout circuits are advantageous even in a VQE scenario where the difference between b_{HT} and b_{TPB} is not negligible. At the beginning of the VQE, the energy of an ansatz state is still high and it is not yet important to estimate this energy with a high resolution. In other words, one can casually operate in the low-shot regime where HT circuits prevail. For example, by decreasing the energy resolution by a factor of 10×, one can save 100× shots (∝ execution cost) during most of the VQE. Eventually, after the VQE has converged, one can still switch from HT to TPB to decrease the final error from b_{HT} to b_{TPB}.

Finally, b_{HT} could be reduced by extending known error mitigation techniques to the case of HT readout circuits [1–3]. We leave this working out the details of this idea as an open problem.

II. GENERAL CLIFFORD CIRCUITS HAVE HUGE OVERHEADS

Our new methodology of *hardware-tailored* (HT) readout circuits interpolates between the concepts of *tensor product bases* (TPBs) and *general Clifford* (GC) circuits, which allow us to measure *qubitwise commuting* (QWC) and *general commuting* (GC) Pauli operators, respectively. Both can be seen as special cases of HT readout circuits, where the connectivity graph Γ_{con} is given by the edgeless (disconnected) graph and the complete (all-to-all connected) graph, respectively. In Fig. 3 of the main text, we have experimentally confirmed that both TPBs and HT readout circuits can be reliably executed on existing quantum hardware. For the same observable (8-qubit hydrogen chain), GC circuits require up to 45 CNOT gates and 78 SWAP gates in a single readout circuit, see Tab. V in SM Sec. XIII. A back-of-the-envelope calculation shows that such a GC diagonalization circuit would have a fidelity on the order of $0.99^{45+3\times78} \approx 0.06$, which renders this option infeasible. Hereby, we assume that each SWAP gate is compiled into three CNOT gates and that every CNOT gate has an error rate on the order of 0.01, see Tab. X in SM Sec. XIII for a justification.

TABLE I. Pauli groupings for different readout methods (meth.) of the n-qubit momentum-space Hubbard Hamiltonian O, see methods section of the main text for details. The hardware connectivity (con.) of TPBs may be arbitrary (arb.). For HT and GC circuits we consider linear (lin.), and cyclic (cyc.) connectivity. The estimated shot reduction $\hat{R}$ is defined in Ref. [4], and N^{circs} denotes the total number of diagonalization circuits to measure all Pauli operators in O. The average and maximum number of CZ gates in the diagonalization circuits are denoted by $N^{\mathrm{avg}}_{\mathrm{CZ}}$ and $N^{\mathrm{max}}_{\mathrm{CZ}}$, respectively. Similarly, $N^{\mathrm{avg}}_{\mathrm{SWAP}}$ and $N^{\mathrm{max}}_{\mathrm{SWAP}}$ stand for the number of SWAP gates that are introduced when we transpile the GC circuits to limited hardware connectivity.

n	M	meth.	con.	$\hat{R}$	N^{circs}	$N^{\mathrm{avg}}_{\mathrm{CZ}}$	$N^{\mathrm{max}}_{\mathrm{CZ}}$	$N^{\mathrm{avg}}_{\mathrm{SWAP}}$	$N^{\mathrm{max}}_{\mathrm{SWAP}}$
6	85	TPB	arb.	3.69	33	0	0	0	0
		HT	lin.	6.58	14	2.64	4	0	0
		HT	cyc.	7.61	11	4.73	6	0	0
		GC	lin.	8.04	10	8	10	15.7	22
		GC	cyc.	8.04	10	8	10	11.8	15
8	166	TPB	arb.	4.45	49	0	0	0	0
		HT	lin.	8.80	21	2.48	6	0	0
		HT	cyc.	8.30	23	3.04	8	0	0
		GC	lin.	13.83	10	24.3	38	45.9	78
		GC	cyc.	13.83	10	24.3	38	35.1	57
10	433	TPB	arb.	3.66	177	0	0	0	0
		HT	lin.	10.55	48	3.81	7	0	0
		HT	cyc.	10.71	47	4.21	9	0	0
		GC	lin.	14.39	29	27.38	48	75.79	115
		GC	cyc.	14.39	29	27.38	48	57.21	108

Similarly, for the ten-qubit Hubbard model, we require up to 48 CZ gates and 115 SWAP gates, see Tab. I. For the same Hamiltonian, HT readout circuits require zero SWAP gates (by design) and up to only seven CZ gates. Note that the TPB and GC groupings in Tab. I are computed with the Sorted Insertion algorithm [4], whereas the HT Pauli groupings are constructed with our Algorithm 1 from SM Sec. V. In Fig. 1, we reproduce a slightly modified version of Fig. 5 **c** from the main text. This time, we plot the estimated shot reduction of grouped Pauli measurements over individual Pauli measurements (and not over TPBs). One can see

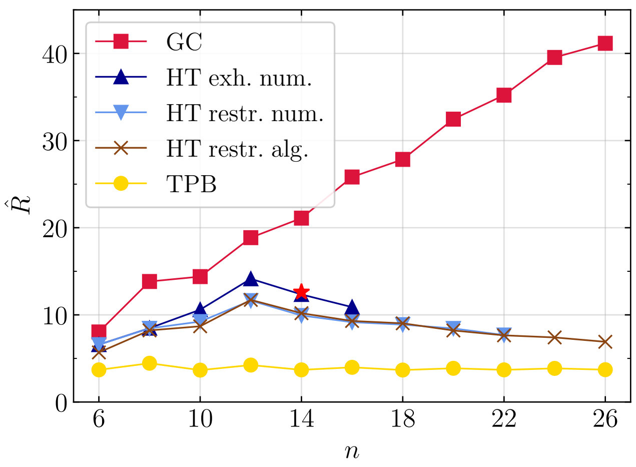

FIG. 1. Estimated shot reduction $\hat{R}$ of various Pauli groupings for one-dimensional, periodic, momentum-space Hubbard Hamiltonians with n-qubit block-spin Jordan-Wigner encoding. The HT readout circuits are constructed with three different solvers as discussed in the methods section of the main text.

that, if GC circuits were a viable option, the available shot reduction $\hat{R}$ would linearly grow with the number of qubits n. For near-term applications, however, our HT circuits give rise to the most efficient readout method that can be executed reliably.

III. COMPLETING A STABILIZER GROUP

The general Clifford (GC) diagonalization circuits constructed in SM Sec. II, have been computed via the algorithm from Ref. [5]. This algorithm, however, only accepts a *maximal* set of commuting Pauli operators as its input. Therefore, we need a subroutine to extend our (non-maximal) sets of commuting Pauli operators. In principle, we could use a well-known approach based on the Gram-Schmidt procedure [6], however, we prefer a custom approach which is described below.

We now explain how to extend a set of m commuting Pauli operators $P_1, \dots, P_m \in \{I, X, Y, Z\}^{\otimes n}$ to a stabilizer group $\mathcal{S}$ of a stabilizer state $|\psi_{\mathcal{S}}\rangle$. Every Pauli operator is of the form $P_i = \mathrm{i}^{q_i} X^{\mathbf{r}^{(i)}} Z^{\mathbf{s}^{(i)}}$ with $q_i \in \{0,1,2,3\}$ and $\mathbf{r}^{(i)}, \mathbf{s}^{(i)} \in \mathbb{F}_2^n$. Since the stabilizer group $\mathcal{S}$ must not contain $-I^{\otimes n}$, some of the operators P_i may have to be replaced by $-P_i$. This does not interfere with our goal because we are only interested in the phase-free part, which is given by the matrix

$$\begin{bmatrix} R \\ S \end{bmatrix} = \begin{bmatrix} \mathbf{r}^{(1)} & \cdots & \mathbf{r}^{(m)} \\ \mathbf{s}^{(1)} & \cdots & \mathbf{s}^{(m)} \end{bmatrix} \in \mathbb{F}_2^{2n \times m}. \tag{1}$$

Using Gaussian elimination over $\mathbb{F}_2$ it is straightforward to find a matrix of row-operations $M \in \mathbb{F}_2^{2n \times 2n}$ such that

$$B = M \begin{bmatrix} R \\ S \end{bmatrix} \tag{2}$$

is the reduced-row echelon form of the matrix in Eq. (1). The non-pivot columns of B correspond to redundant Pauli operators P_i that can be written as products of other operators in the set $\{P_1, \dots, P_m\}$. To simplify our notation and the complexity of the algorithm, we remove the redundant operators and, by slight abuse of notation, call the remaining operators $P_1, \dots, P_m$, which are now independent in the sense that the matrix B in Eq. (2) does not contain any non-pivot columns anymore, i.e.,

$$B = \begin{bmatrix} \mathbb{1}_m \\ 0 \end{bmatrix} \in \mathbb{F}_2^{2n \times m}. \tag{3}$$

The intricate part of completing these operators to $\mathcal{S}$ is to find an operator $P_{m+1} \in \{I, X, Y, Z\}^{\otimes n}$ that simultaneously is independent from and commuting with all previous operators. By the symplectic nature of the Pauli group, an operator $X^{\mathbf{j}} Z^{\mathbf{k}}$ commutes with $P_1, \dots, P_m$ if and only if (iff)

$$\begin{bmatrix} R \\ S \end{bmatrix}^{\mathrm{T}} \begin{bmatrix} 0 & \mathbb{1}_n \\ \mathbb{1}_n & 0 \end{bmatrix} \begin{bmatrix} \mathbf{j} \\ \mathbf{k} \end{bmatrix} = 0, \tag{4}$$

i.e., iff $[\mathbf{j}, \mathbf{k}] \in \ker([S^{\mathrm{T}}, R^{\mathrm{T}}])$ [7]. Furthermore, $X^{\mathbf{j}} Z^{\mathbf{k}}$ is independent from $P_1, \dots, P_m$ iff appending the vector $[\mathbf{j}, \mathbf{k}]^{\mathrm{T}}$ as a final column to the matrix in Eq. (1) increases the rank by one. Looking at Eq. (3), we find that this is the case iff the vector $\mathbf{v} = M[\mathbf{j}, \mathbf{k}]^{\mathrm{T}}$ fulfills $v_i = 1$ for at least one index $i > m$. Therefore, the set of binary vectors that are independent of $[\mathbf{r}^{(1)}, \mathbf{s}^{(1)}], \dots, [\mathbf{r}^{(m)}, \mathbf{s}^{(m)}]$ is given by $M^{-1}\mathcal{L}$, where

$$\mathcal{L} = \left\{ \mathbf{v} \in \mathbb{F}_2^{2n} \;\middle|\; \exists i > m : v_i = 1 \right\}. \tag{5}$$

Combining both properties, we need to find a vector in the intersection

$$\ker([S^{\mathrm{T}}, R^{\mathrm{T}}]) \cap M^{-1}\mathcal{L} = M^{-1} \left(\ker([S^{\mathrm{T}}, R^{\mathrm{T}}] M^{-1}) \cap \mathcal{L} \right).$$

To achieve this, we first apply Gaussian elimination to compute a basis $\mathbf{v}^{(1)}, \dots, \mathbf{v}^{(2n-m)} \in \mathbb{F}_2^{2n}$ of the null space of the matrix

$$\begin{bmatrix} S \\ R \end{bmatrix}^{\mathrm{T}} M^{-1} \in \mathbb{F}_2^{m \times 2n}. \tag{6}$$

Next, we select a vector $\mathbf{v}^{(i)}$ with $v_j^{(i)} = 1$ for at least one index $j > m$ (which exists because of $m < n$). In particular, we have $\mathbf{v}^{(i)} \in \ker([S^{\mathrm{T}}, R^{\mathrm{T}}] M^{-1}) \cap \mathcal{L}$ and Eq. (6) shows that $P_{m+1} = \pm X^{\mathbf{r}^{(m+1)}} Z^{\mathbf{s}^{(m+1)}}$ with

$$\begin{bmatrix} \mathbf{r}^{(m+1)} \\ \mathbf{s}^{(m+1)} \end{bmatrix} = M^{-1} \mathbf{v}^{(i)} \tag{7}$$

extends the set $\{P_1, \dots, P_m\}$ in the desired way. We can repeat all these steps until we finally reach $m = n$.

IV. OPTIMALLY ALLOCATING SHOTS TO READOUT CIRCUITS

In this section, we review the well-known strategy for optimally distributing a fixed number N^{shots} of shots among the readout circuits of a given Pauli grouping [4]. Building on this, we then propose a shot-allocation heuristic, which does not require any knowledge about the state that is measured. This supplementary note is intended to motivate Eq. (15) in SM Sec. V, which was used for the construction of the hardware-tailored Pauli groupings throughout this work.

Consider a decomposition $O = \sum_{i=1}^{M} c_i P_i = \sum_{i=1}^{N} O_i$ into fragments $O_i = \sum_{j=1}^{m_i} c_{i,j} P_{i,j}$, for each of which the Pauli operators $P_{i,1}, \ldots, P_{i,m_i}$ can be measured with a single readout circuit. For a fixed shot budget, it is important to know how the number of available shots should be distributed over the different measurement circuits. The selected shot allocation has an influence on the resulting estimator $\widehat{\langle O \rangle}$ of the true expectation value $\langle O \rangle$. The optimal shot allocation minimizes the variance $\epsilon^2 = \text{Var}[\widehat{\langle O \rangle}]$ under the constraint $N^{\text{shots}} = \sum_{i=1}^{N} n_i$, where n_i denotes the number of shots assigned for the measurement of fragment O_i. In an experiment, the samples for measuring different fragments are obtained independently if we restrict ourselves to the paradigm of non-overlapping Pauli groupings [8]. This manifests itself in $\text{Cov}[\widehat{\langle O_i \rangle}, \widehat{\langle O_j \rangle}] = 0$ for all $i \neq j$. Thus, we obtain

$$\epsilon^2 = \sum_{i=1}^{N} \text{Var}\left[\widehat{\langle O_i \rangle}\right]. \tag{8}$$

Since the variances $\text{Var}[\widehat{\langle O_i \rangle}] = \text{Var}[O_i]/n_i$ directly follow from the standard error on the sample mean, we find

$$\epsilon^2 = \sum_{i=1}^{N} \frac{1}{n_i} \sum_{j,j'=1}^{m_i} c_{i,j} c_{i,j'} \text{Cov}\left[P_{i,j}, P_{i,j'}\right]. \tag{9}$$

It is therefore evident that the covariances of the Pauli operators within a given fragment can strongly influence the final accuracy [9].

In Ref. [4], Crawford et al. exploit Lagrange multipliers to show that Eq. (9) is minimized for

$$n_i \propto \sqrt{\text{Var}[O_i]}, \tag{10}$$

where the proportionality constant is given by

$$\frac{N^{\text{shots}}}{\sum_{j=1}^{N} \sqrt{\text{Var}[O_j]}} = \frac{1}{\epsilon^2} \sum_{j=1}^{N} \sqrt{\text{Var}[O_j]}. \tag{11}$$

This provides the optimal strategy for distributing the available shots among the different readout circuits. Implementing this strategy in practice, however, requires knowledge about $\text{Var}[O_1], \ldots, \text{Var}[O_N]$. There exist several approaches to address this problem: one possibility is to approximate these variances by performing a classically tractable calculation [10]. For certain quantum algorithms, one can exploit an adaptive shot-allocation strategy [11–13]. Finally, one can experimentally obtain an estimator $\widehat{\text{Var}[O_i]}$ from the same measurement data that is gathered for the estimation of $\widehat{\langle O_i \rangle}$ [5].

When implementing the latter approach, one has to acquire data by executing the measurement circuit for O_i. In the beginning, the optimal shot allocation is still unknown since any information about $\text{Var}[O_i]$ is not yet available. Thus, one has to rely on heuristics, such as simply allocating the same number of shots to each of the circuits. As an alternative, we introduce a slightly more sophisticated shot-allocation heuristic. Writing out Eq. (10) yields

$$n_i = N^{\text{shots}} \frac{\sqrt{\sum_{j,j'=1}^{m_i} c_{i,j} c_{i,j'} \text{Cov}(P_{i,j} P_{i,j'})}}{\sum_{k=1}^{N} \sqrt{\sum_{j,j'=1}^{m_k} c_{k,j} c_{k,j'} \text{Cov}(P_{k,j}, P_{k,j'})}}. \tag{12}$$

Borrowing an idea from Ref. [4], we replace the covariances in Eq. (12) by their average over the spherical measure, which is given by $\mathbb{E}[\text{Cov}[P, P']] = \delta_{P,P'}/(1 + 2^{-n})$ for all commuting operators $P, P' \in \{I, X, Y, Z\}^{\otimes n}$ (see Ref. [5, Thrm. 3] and Ref. [4, Eq. (45)]). This yields our a-priori shot-allocation heuristic, which only depends on the observable O and its Pauli grouping,

$$n_i^{\text{heuristic}} = \frac{N^{\text{shots}} \sqrt{m_i \sum_{j=1}^{m_i} c_{i,j}^2}}{\sum_{k=1}^{N} \sqrt{m_k \sum_{j=1}^{m_k} c_{k,j}^2}}. \tag{13}$$

Note that Eq. (13) recovers the asymptotically-optimal shot allocation $n_i \propto |c_i|$ in the ungrouped case [14, 15]. We leave investigating the performance of our heuristic shot allocation strategy as an open problem. For other, more recent developments on allocating shots to readout circuits, see Refs. [16, 17].

V. PAULI GROUPING ALGORITHMS

In quantum simulation experiments, it is necessary to estimate the expectation value $\langle O \rangle$ of a given observable O [18]. This can be accomplished, for instance, by first measuring all Pauli operators P_i that occur in $O = \sum_{i=1}^{M} c_i P_i$ and computing $\langle O \rangle = \sum_{i=1}^{M} c_i \langle P_i \rangle$ in a classical post-processing step. Here, the first step can be carried out more efficiently if multiple Pauli operators are measured simultaneously. Prior to an experiment, it is thus desirable to group $P_1, \ldots, P_M$ into jointly-measurable collections; we refer to this challenge as the Pauli-grouping problem. In July 2019,

three groups of researchers pointed out in a number of preprints (arXiv:1907.03358, arXiv:1907.07859, arXiv:1907.09386, and arXiv:1907.13623) that the Pauli-grouping problem is equivalent to the clique-cover problem of the commutativity graph of O and, dually, to the coloring problem of the non-commutativity graph of O [5, 19–21]. This important insight implies that the number N of jointly-measurable collections required for measuring all operators in O is lower-bounded by the chromatic number of the non-commutativity graph of O. Furthermore, groupings that minimize N were investigated in these seminal papers. Later, it was recognized that $\langle O \rangle$ can be estimated even more efficiently if not only N is kept small, but also if the coefficients c_i in the decomposition of O are taken into account [4].

In this section, we adapt the *Sorted Insertion* (SI) algorithm of Ref. [4] to our purposes. Thus, we briefly review it now. First, the Pauli operators $P_1, \dots, P_M$ are *sorted* such that $|c_1| \geq |c_2| \geq \dots \geq |c_M|$. Then, P_1 is assigned to a first jointly-measurable collection. If P_2 commutes with P_1, it is assigned to this first collection as well; otherwise, a second collection is created. Similarly, one proceeds with $j \in \{3, \dots, M\}$ by iterating through the existing collections until one is found into which P_j can be *inserted*. In the original version of SI, the insertion condition is given by *general commutativity* (GC). Accordingly, each of the resulting collections can, in principle, be measured by applying a diagonalization circuit, followed by a readout of the individual qubits. In general, however, these circuits are too demanding for near-term applications as discussed in SM Sec. II. To remedy this deficiency, one can replace the GC condition by *qubitwise commutativity* (QWC). We refer to this minor modification as SI-QWC. The corresponding readout circuits are minimal in their hardware demands; however, only tensor product bases can be measured.

By integrating our approach for the construction of *hardware-tailored* (HT) readout circuits, we can improve upon SI-QWC without sacrificing hardware-efficiency. As a straightforward modification of SI, one could simply replace the GC insertion condition by HT-measurability. For the HT Pauli groupings computed throughout this work (unless specified otherwise), however, we implement Algorithm 1, which has the advantage of parallel executability. As in SI, we sort the operators $P_1, \dots, P_M$ by the magnitude of their coefficients. In every iteration of the outer loop (lines 4–22), which is repeated until all operators have been assigned to a jointly-HT-measurable collection, we construct a readout circuit for the operator P_{main} (line 5) that is leading the list of still-unassigned, remaining Pauli operators. Hereby, we strive for a readout circuit that also works for other remaining Pauli operators. To find the circuit with the "best" collection, we loop over a prespecified list of $s(n)$ subgraphs Γ of the connectivity graph Γ_{con} (lines 7–18); this can be executed in parallel. For every subgraph Γ, we first attempt the construction of a Γ-based circuit (see main text, Fig. 1) that diagonalizes P_{main} (line 9). For this, we exploit one of the solvers from Tab. III of the main text. By varying the hyperparameters of the selected solver, one can flexibly adjust Algorithm 1; we explore this possibility in Fig. 5 of the main text and in SM Secs. VII and XIV. If, for a given Γ, the solver succeeds in constructing a HT readout circuit for P_{main}, we start a collection (line 10) of operators that can be measured with a circuit based on this instance of Γ. Then, we successively extend this collection by iterating through the list of remaining operators (lines 11–15) and adding those Pauli operators which can be measured with a Γ-based readout circuit as well. Hereby, we allow updating the single-qubit Clifford layer $U_1 \otimes \dots \otimes U_n$ of the Γ-based readout circuit.

Algorithm1

Parallel algorithm for grouping a list of Pauli operators, `paulis`, into a list of collections, `out`. Every collection in `out` can be measured with a *hardware-tailored* (HT) readout circuit as in Fig. 1 of the main text. Hereby, a pre-defined list of `subgraphs` specifies templates for the readout circuits. Note that `subgraphs` must contain $\Gamma = 0$ to ensure that the algorithm will terminate. As in Sorted Insertion [4], the operators in `paulis` are sorted with respect to their coefficients. To construct HT circuits, any solver from Tab. III of the main text can be exploited. If, for a given subgraph Γ and a collection `col` of Pauli operators, the selected solver succeeds in constructing a circuit, we write `ht_measurable(col, Γ) = True`.

```
1: out ← ∅;
2: s ← length(subgraphs);
3: remaining_paulis ← paulis;
4: while remaining_paulis ≠ ∅ do
5:    main_pauli ← remaining_paulis[0];
6:    temp_collections ← [∅, ..., ∅];          ▷ list of length s
7:    for i in [0, ..., s-1] do                 ▷ parallel loop
8:       Γ ← subgraphs[i];
9:       if ht_measurable([main_pauli], Γ) then
10:         col ← [main_pauli];
11:         for pauli in remaining_paulis do
12:            if ht_measurable(col ∪ [pauli], Γ) then
13:               col ← col ∪ [pauli];
14:            end if
15:         end for
16:         temp_collections[i] ← col;
17:      end if
18:   end for
19:   best_collection ← best(temp_collections);
20:   remaining_paulis.remove(best_collection);
21:   out.append(best_collection);
22: end while
23: return out;
```

At this point, Algorithm 1 can be sped up by checking if Eq. (6) of the main text, by chance, holds for the previous single-qubit Clifford layer and the new Pauli operator. In general, however, new single-qubit Clifford layers are computed by reapplying the solver to the growing collection of operators. This step could likely benefit from a warm starting method; we leave this as an open problem. Since the list of remaining Pauli operators is sorted, the collection prioritizes operators P_i for which $|c_i|$ is large; this idea is borrowed from Ref. [4]. By doing all of this, we create one collection for every subgraph Γ under consideration. For those subgraphs Γ for which P_{main} does not admit a Γ-based readout circuit (or the solver fails finding it), the corresponding collection stays empty (line 6), i.e., we simply skip Γ. Among the list of the constructed collections $\{P_{i_1}, \ldots, P_{i_m}\}$ containing $P_{i_1} \equiv P_{\text{main}}$, we regard the one which maximizes a suitable value function, e.g.,

$$\text{value}_1(\{P_{i_1}, \ldots, P_{i_m}\}) = m \tag{14}$$

$$\text{or} \quad \text{value}_2(\{P_{i_1}, \ldots, P_{i_m}\}) = m \sum_{k=1}^{m} c_{i_k}^2 \tag{15}$$

as the best collection (line 19). In the final Pauli grouping produced by Algorithm 1, the operator P_{main} has to occur only once; we select the best collection (line 21). Finally, we remove the operators in the best collection from the list of remaining Pauli operators (line 20). All of these steps are repeated until all operators have been grouped into a jointly-HT-measurable collection. To ensure that Algorithm 1 will terminate, it is of crucial importance to include the trivial subgraph $\Gamma = 0$. Hereby, one should simply test if the Pauli operators $P_{i_1}, \ldots, P_{i_m}$ are QWC (and not employ any of the solvers from Tab. III of the main text).

The efficiency of Algorithm 1 depends on the solver that is exploited for constructing the readout circuits. In Fig. 5 of the main text, we demonstrate that the `restricted algebraic solver` from Tab. III of the main text can construct enough readout circuits to ensure both a polynomial runtime and a high-quality HT Pauli grouping. While collecting the data for Fig. 5 of the main text, we discovered a potentially useful fact: in the beginning, the ("best") collections are comparatively large, but over the course of Algorithm 1 they often become smaller and smaller. Since the important Pauli operators P_i, for which $|c_i|$ is large, are assigned first, we expect that a considerable amount of computational time can be saved (without greatly deteriorating the result) if, eventually, SI-QWC takes over from Algorithm 1. Investigating such ideas is beyond the scope of this work but deserves further consideration.

Note that the objective function in Eq. (15), whose definition is motivated by the enumerator of Eq. (10) in SM Sec. IV, slightly outperforms the one in Eq. (14) (see Tab. II in SM Sec. VII). In future research, one could study in more detail how the quality of HT Pauli groupings is impacted by the choice of these and other objective functions, e.g., one that penalizes low-fidelity CZ gates. Another promising use case for such objective functions is the exploration of the set of subgraphs: so far, we simply iterate through a prespecified subset of subgraphs $\Gamma \subset \Gamma_{\text{con}}$. It is worth investigating if this can be made more efficient, e.g., via simulated annealing or Monte Carlo methods.

VI. PREPROCESSING TIME VS. RUNTIME SAVINGS FOR A 52-QUBIT EXAMPLE

In this section, we demonstrate the possibility to scale up the construction of hardware-tailored (HT) readout circuits to problem sizes that are beyond the border of classical simulatability. The sole purpose of this section is to establish that our new methods *can* be scaled up to large systems. For any practical application, we stress the importance of further developing the methods presented here. In this way, one will likely reach much better savings than the ones predicted in the proof-of-principle example below. Nevertheless, we will see that the costs for classically precomputing HT circuits can be small compared to the enabled quantum runtime savings.

We consider a linear chain with $L = 26$ hydrogen atoms at an interatomic distance of $d = 1.0$ Å. Here, we express this molecule in the STO-3G basis set and use the *Bravyi-Kitaev* (BK) mapping to obtain a Hamiltonian $O = \sum_{i=1}^{M} c_i P_i$ with $M = 443,715$ Pauli operators on $n = 52$ qubits. First, we apply SI-QWC (see SM Sec. V) to group $P_1, \ldots, P_M$ into $N_{\text{TPB}} = 136,325$ *tensor product bases* (TPBs) as this represents the prior state of the art upon which we will improve. While the average number of $M/N_{\text{TPB}} \approx 3.25$ Pauli operators per TPB is consistent with previous observations [19], we stress that the operators are far from being evenly distributed among the TPBs. In Fig. 2, we show that the number of TPBs containing a given number m_i of Pauli operators follows a distribution (green dots) which is well approximated by a power-law decay of $10^5 \times m_i^{-2}$ (gray line). Importantly, there are $70,579$ Pauli operators with $m_i = 1$, which are mutually non-QWC. Hence, each of them requires its own TPB, i.e., if we want to group these "ungrouped" operators into jointly-measurable sets, we need to permit two-qubit gates in the readout circuits. Note that for BK Hamiltonians the weight (size of the support) of the involved Pauli operators P_i only grows logarithmically in n [22]. We exploit this fact in Algorithm 2.

The underlying principle of Algorithm 2 is the same as that of Algorithm 1 from Sec. V: in every iteration of the outer loop (lines 3-26), we construct a HT readout circuit for P_{main}, which is leading the list $\mathcal{P}_{\text{rem}}$ of remaining Pauli operators. This time, we remove all operators from $\mathcal{P}_{\text{rem}}$ that do not commute with P_{main} because none of them is jointly-HT-

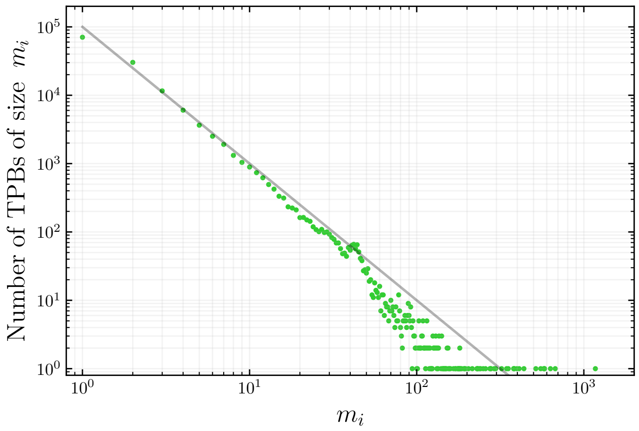

FIG. 2. Size distribution of the $N_{\text{TPB}} = 136,325$ *tensor product bases* (TPBs) into which the $M = 443,715$ Pauli operators of a Bravyi-Kitaev Hamiltonian with $n = 52$ qubits can be grouped. The Hamiltonian describes a linear hydrogen chain with $L = 26$ sites. Whereas there exists only one TPB that contains as many as $m_i = 1,172$ operators, most TPBs (70,579 in total) only contain a single Pauli operator.

measurable with P_{main}; the resulting selection is denoted by $\mathcal{P}_{\text{sel}} = f_1(\mathcal{P}_{\text{rem}}, P_{\text{main}})$ (line 5). Our new idea is to restrict to subgraphs of $\Gamma|_{\text{supp}(P_{\text{main}})}$, i.e., only two-qubit gates within the support of P_{main} are allowed. In this way, the efficiency of Algorithm 2 is ensured because of $|\text{supp}(P_i)| = \mathcal{O}(\log(n))$ for all $i \in \{1, \ldots, M\}$. Naively, one might try to proceed by only taking the operators $P_i \in \mathcal{P}_{\text{sel}}$ with $\text{supp}(P_i) \subset \text{supp}(P_{\text{main}})$ into account as this would render the measurements outside of the support of P_{main} irrelevant and consequently speed up the construction of readout circuits; however, such operators are prohibitively rare. As a more sophisticated approach, we instead guess a suitable single-qubit measurement basis for every qubit $j \in \{1, \ldots, n\} \backslash \text{supp}(P_{\text{main}})$. Our guess is the single-qubit Pauli operator $P_{\text{mf}}^{(j)} \in \{X, Y, Z\}$ that occurs *most frequently* among the n-qubit Pauli operators $P = P^{(1)} \otimes \ldots \otimes P^{(n)} \in \mathcal{P}_{\text{sel}}$ on qubit j, i.e.,

$$P_{\text{mf}}^{(j)} = \underset{p \in \{X,Y,Z\}}{\arg\max} \left| \left\{ P \in \mathcal{P}_{\text{sel}} \;\middle|\; P^{(j)} = p \right\} \right| . \tag{16}$$

Next, we refine our selection $\mathcal{P}_{\text{sel}}$ of considered Pauli operators by filtering out all of those that cannot be measured for this guess (line 7), which results in the set

$$f_2(\mathcal{P}_{\text{sel}}, J) = \left\{ P \in \mathcal{P}_{\text{sel}} \;\middle|\; \forall j \in J^{\text{C}} : P^{(j)} \in \{I, P_{\text{mf}}^{(j)}\} \right\}, \tag{17}$$

where $J^{\text{C}} = \{1, \ldots, n\} \backslash J$ denotes the complement of $J = \text{supp}(P_{\text{main}})$. Note that for our purposes $f_2(\mathcal{P}_{\text{sel}}, J)$ is sufficiently large, e.g., in the first iteration of Algorithm 2 applied to the set of $|\mathcal{P}_{\text{rem}}| = 70,579$ operators that remained "ungrouped" after applying SI-QWC to the 52-qubit hydrogen-chain Hamiltonian, we still have $|f_2(\mathcal{P}_{\text{sel}}, J)| = 31$ operators remaining (for $m_1^{\text{HT}} = 5$ of them, Algorithm 2 is able to construct a common HT readout circuit). Then, we proceed by making a selection of subgraphs $\Gamma \subset \Gamma|_{\text{supp}(P_{\text{main}})}$ that will be considered as circuit templates (line 8). Hereby, one can use either all such subgraphs or a random selection of up to $s_{\max}$ (a hyperparameter) of them. The remaining steps are the same as for Algorithm 1, with the exception that this time we only attempt to include operators from our filtered selection $f_2(\mathcal{P}_{\text{sel}}, J)$ (line 15).

Algorithm2

Modification of Algorithm 1 in SM Sec. V. This variant is efficient if the support of the operators in `paulis` only grows logarithmically in the number n of qubits, e.g., for Bravyi-Kitaev Hamiltonians.

```
1:  out ← ∅;
2:  remaining_paulis ← paulis;
3:  while remaining_paulis ≠ ∅ do
4:     main_pauli ← remaining_paulis[0];
5:     selection ← f1(remaining_paulis, main_pauli);
6:     main_support ← support(main_pauli);
7:     selection ← f2(selection, main_support);
8:     subgraphs ← select_subgraphs(main_support);
9:     s ← length(subgraphs);
10:    temp_collections ← [∅, ..., ∅];          ▷ list of length s
11:    for i in [0, ..., s-1] do                 ▷ parallel loop
12:       Γ ← subgraphs[i];
13:       if ht_measurable([main_pauli], Γ) then
14:          col ← [main_pauli];
15:          for pauli in selection do
16:             if ht_measurable(col ∪ [pauli], Γ) then
17:                col ← col ∪ [pauli];
18:             end if
19:          end for
20:          temp_collections[i] ← col;
21:       end if
22:    end for
23:    best_collection ← best(temp_collections);
24:    remaining_paulis.remove(best_collection);
25:    out.append(best_collection);
26: end while
27: return out;
```

We apply Algorithm 2 to the aforementioned set of $70,579$ "ungrouped" 52-qubit Pauli operators and keep track of its performance over a period of two and a half weeks, see Fig. 3. In total, we group $8,231$ Pauli operators into $3,935$ jointly-HT-measurable sets, where a two-dimensional hardware connectivity with up to four nearest neighbors is assumed. For every $N \in \{1, \ldots, 3935\}$ when the algorithm identifies the N-th set of jointly-HT-measurable Pauli operators (line 25) we extract: the current average number $\overline{m}_N^{\text{HT}} = (m_1^{\text{HT}} + \ldots + m_N^{\text{HT}})/N$ of operators per

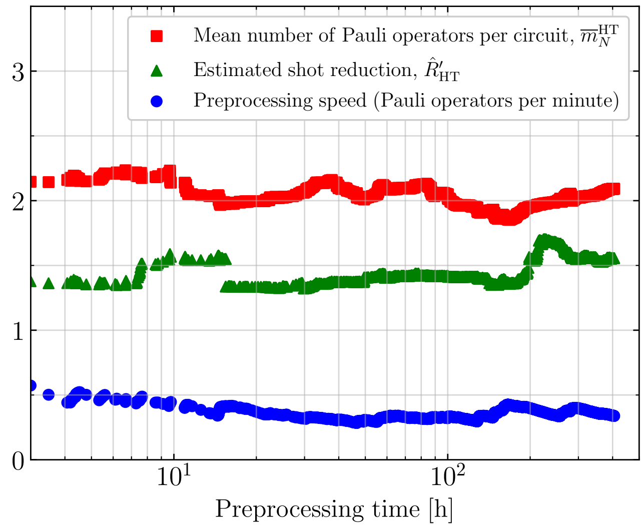

FIG. 3. Performance of Algorithm 2 when applied to the list of 70,579 Pauli operators from Fig. 2 with $m_i = 1$. We use the `restricted algebraic solver` from Tab. III in the main text with a cutoff of $c = 5$ and tailor the readout circuits to a 2D square-lattice connectivity, taking up to $s = 5,000$ subgraphs into account (here: not in parallel for simplicity). All computations were carried out on an 18 core Intel Xeon CPU E5-2697 v4 @2.30GHz device.

readout circuit (red squares), the estimated reduction $\hat{R}'_{\mathrm{HT}}$ in the number of shots needed to measure the current partial observable $O' = \sum_{i=1}^{N} \sum_{j=1}^{m_i^{\mathrm{HT}}} c_{i,j} P_{i,j}$ (green triangles), and the current preprocessing speed $(m_1^{\mathrm{HT}} + \ldots + m_N^{\mathrm{HT}})/t_N$ at which the Pauli operators are being grouped (blue circles). Then, we plot these three quantities as a function of the time t_N after which the N-th readout circuit has been assigned (line 25). On average, we find $\overline{m}_N^{\mathrm{HT}} \approx 2$ Pauli operators per jointly-HT-measurable collection ($\min m_N^{\mathrm{HT}} = 1$, $\max m_N^{\mathrm{HT}} = 11$) and an associated shot reduction of $\hat{R}_{\mathrm{HT}} \approx 1.5$. Note that $\hat{R}_{\mathrm{TPB}} = 1$ for the considered problem. The observed average speed of the grouping algorithm is equal to almost one Pauli operator every two minutes; note that we did not parallelize the loop over the subgraphs (lines 11–22) to reduce our implementation efforts for this proof-of-principle demonstration. For practical applications, however, we highly recommend a parallel implementation as this would significantly enhance the classical preprocessing speed. Our most important insight from Fig. 3 is that the performance of Algorithm 2 stays stable over long periods of time. Even if, at a later stage, the performance should suddenly deteriorate, one could simply terminate Algorithm 2 at any time and decide to individually measure the remaining Pauli operators in their respective TPBs.

Next, we show that for performances as in Fig. 3, it pays off to execute Algorithm 2. For a given observable $O = \sum_{i=1}^{M} c_i P_i$, the runtime that is saved on the quantum computer through the availability of a HT Pauli grouping of any *subset* of the set $\{P_1, \ldots, P_M\}$ can be assessed as

$$T_{\mathrm{saved}} = T_{\mathrm{shot}} \times N_{\mathrm{saved\ shots/est.}} \times N_{\mathrm{est.}}, \tag{18}$$

where T_{shot} is the time needed for the execution of a single quantum circuit ("1 shot"), $N_{\mathrm{est.}}$ is the number of experimental trial states ρ for which the expectation value $\mathrm{Tr}[O\rho]$ is estimated, and $N_{\mathrm{saved\ shots/est.}}$ denotes the number of quantum circuit executions that can be avoided (whilst maintaining a given accuracy) for such an estimation if the grouped Pauli operators are measured with HT readout circuits instead of TPBs. On current IBM superconducting quantum processors, the duration of a two-qubit gate is on the order of 500 ns, see Tab. X in Sec. XIII. If the ansatz quantum circuit for the preparation of ρ relies on the implementation of gates of the form $\exp(icP)$ with $c \in \mathbb{R}$, $P \in \{I, X, Y, Z\}^{\otimes n}$ as in Sec. IX, the required number of two-qubit gate layers (circuit depth) can be well above 100. In this case we find

$$T_{\mathrm{shot}} > 50\,\mu\mathrm{s}, \tag{19}$$

which is within the window of coherence times reported in Tab. X. Assume that we have grouped the first $M' < M$ Pauli operators into jointly-HT-measurable subsets. The estimated relative number of saved shots (assumed to be allocated optimally, see Sec. IV) for measuring the partial observable $O' = \sum_{i=1}^{M'} c_i P_i$ with HT readout circuits instead of TPBs is given by $\hat{R}'_{\mathrm{HT}}/\hat{R}'_{\mathrm{TPB}}$ [4]. Hence, the total number of shots saved per estimation of $\mathrm{Tr}[O'\rho]$ follows as

$$N_{\mathrm{saved\ shots/est.}} = \left(1 - \hat{R}'_{\mathrm{TPB}}/\hat{R}'_{\mathrm{HT}}\right) N'_{\mathrm{alloc.shots}}, \tag{20}$$

where $N'_{\mathrm{alloc.shots}}$ denotes the number of shots that we would have allocated to the measurement of $P_1, \ldots, P_{M'}$ in a TPB approach. The number of iterations that are needed before the variational quantum eigensolver (VQE) algorithm starts to converge is at least on the order of $N_{\mathrm{converge}} = 100$ [23]. By running the VQE algorithm once for each of $N_{\mathrm{pos.}}$ positions of the nuclei of a molecule, one can map out its dissociation curve without the need to reconstruct any HT readout circuits as we discuss around Fig. 4 of the main text. This can be repeated for the first few excited states or "energy bands" whose number we denote by N_{bands}. Finally, one might want to compare the results for $N_{\mathrm{ansätze}}$ differently parameterized ansatz circuit architectures. Assuming $N_{\mathrm{pos.}} = 100$, $N_{\mathrm{bands}} = 10$, and $N_{\mathrm{ansätze}} = 10$, the number of different states ρ for which $\mathrm{Tr}[O\rho]$ needs to be estimated in such a scenario is given by

$$\begin{aligned} N_{\mathrm{est.}} &= N_{\mathrm{converge}} \times N_{\mathrm{pos.}} \times N_{\mathrm{bands}} \times N_{\mathrm{ansätze}} \\ &= 1,000,000. \end{aligned} \tag{21}$$

Other use cases that require an enormously large number $N_{\text{est.}}$ of estimations include the simulation of quantum dynamics, where $\text{Tr}[O\rho(t)]$ is mapped out for a time-series of quantum states $\rho(t)$, as well as simulations of chemical reactions.

Let us derive an estimate of how large $N_{\text{est.}}$ needs to be before the construction of the 3,935 HT readout circuits for the $M' = 8,231$ Pauli operators from Fig. 3 pays off. For concreteness, we assume $N'_{\text{alloc.shots}} = 823,100$, i.e., every Pauli expectation value is estimated with an average number of 100 shots. Inserting this together with $\hat{R}'_{\text{HT}} = 1.56$ and $\hat{R}'_{\text{TPB}} = 1$ into Eq. (20) yields

$$N_{\text{saved shots/est.}} \approx 300,000. \tag{22}$$

Combining this with Eq. (19) shows that we would save about 1.5 seconds for every state ρ for which we estimate $\text{Tr}[O\rho]$. On the other hand, the preprocessing time needed on the classical computer for constructing the HT circuits was slightly less than $T_{\text{proc.}} = 1.5 \times 10^6$ seconds. In the extensive scenario described in Eq. (21), we would thus save as much time on the quantum computer (neglecting times waiting in the queue before the quantum circuits are executed) as we need to invest on the classical computer (in principle, in parallel). However, time is not the only resource that should be taken into consideration. Accessing one 127-qubit IBM Eagle quantum processor is commercially possible for a charge of \$1.60 per runtime second [24]. In view of the modest current CPU hourly rates of about \$0.10, the construction of HT readout circuits already pays off (in terms of money) after as little as $N_{\text{est.}} \approx 200$ usages. Again, let us stress the importance of the reusability potential of HT Pauli groupings.

While this side-by-side comparison already demonstrates the financial viability of our method in its current form, we expect that further theoretical improvements can significantly enhance the advantage of HT readout circuits over TPBs in the future.

Finally, note that T_{shot}, and therefore also T_{saved}, is typically three orders of magnitude larger for ion-trap or neutral-atom quantum computers. Hence, for these technologies the break-even of classical preprocessing time and saved time on the quantum computer would occur much earlier.

VII. EXPLORING HYPERPARAMETER CHOICES FOR MOLECULAR HAMILTONIANS

In this section, we explore how the choice of hyperparameters influences the performance of Algorithm 1 from Sec. V. At the same time, we demonstrate that the usefulness of our new HT circuits extends to even more example classes than we already covered in Fig. 5 of the main text.

Choice of molecules

We consider the following selection of molecules:

- LiH with an interatomic distance of 1.545 Å.
- H_4 on a 1.7380 Å × 1.5148 Å rectangular geometry.
- N_2 with an interatomic distance of 1.25 Å.
- H_2O with oxygen at the origin and the two hydrogen nuclei on the $z = 0$ plane with coordinates $x_1 = 0$, $y_1 = 0.591$ Å and $x_2 = 0.572144$ Å, $y_2 = -0.148094$ Å.
- HCN on a linear arrangement along the x-axis with $x_{\text{H}} = -1.10$ Å, $x_{\text{C}} = 0$, and $x_{\text{N}} = 1.15$ Å.

For LiH, we can take the Pauli decomposition of the second-quantized molecular Hamiltonian

$$\hat{H} = \sum_{p,q} h_{p,q} \hat{a}_p^\dagger \hat{a}_q + \frac{1}{2} \sum_{p,q,r,s} h_{p,q,r,s} \hat{a}_p^\dagger \hat{a}_q^\dagger \hat{a}_r \hat{a}_s, \tag{23}$$

directly from Table S2 of Ref. [23]. Note that both sums in Eq. (23) run over all combinations of considered molecular orbitals ϕ_p with creation operator $\hat{a}_p^\dagger$ [18]. For the other molecules, we perform restricted Hartree-Fock calculations using PySCF [25], which yields the one-body integrals

$$h_{p,q} = \int \mathrm{d}\mathbf{x} \phi_p^*(\mathbf{x}) \left(-\frac{\nabla^2}{2} - \sum_I \frac{Z_I}{|\mathbf{r} - \mathbf{R}_I|} \right) \phi_q(\mathbf{x}), \tag{24}$$

(where $\mathbf{x} = (\mathbf{r}, \sigma)$ denotes position $\mathbf{r}$ and spin σ of an electron, and Z_I and $\mathbf{R}_I$ are atomic number and position of the I-th nucleus), as well as the two-body integrals

$$h_{p,q,r,s} = \int \mathrm{d}\mathbf{x}_1 \mathrm{d}\mathbf{x}_2 \frac{\phi_p^*(\mathbf{x}_1)\phi_q^*(\mathbf{x}_2)\phi_r(\mathbf{x}_2)\phi_s(\mathbf{x}_1)}{|\mathbf{r}_1 - \mathbf{r}_2|}. \tag{25}$$

For simplicity, here we consider the STO-3G minimal basis set $\{\phi_p\}$. Note, however, that our methods also work in the case of more sophisticated basis sets, see Sec. XIV. The fermionic operators are mapped to Pauli operators using the parity encoding [22], where we make use of precision-preserving qubit-reduction methods as proposed in Refs. [26–28] and implemented in Qiskit [29]. This leads to Hamiltonians whose numbers n and M of qubits and Pauli operators, respectively, are provided in the left part of Tab. II.

We apply various Pauli grouping algorithms to the molecular Hamiltonians from above and present the quality of the result in Tab. II. As expected, we observe the relationship $\hat{R}_{\text{TPB}} < \hat{R}_{\text{HT}} < \hat{R}_{\text{GC}}$. Recall from SM Sec. II that $\hat{R}_{\text{GC}}$ is inaccessible with near-term quantum hardware. Hence, the improvement of HT readout circuits over TPBs which ranges from $\hat{R}_{\text{HT}}^{(15)}/\hat{R}_{\text{TPB}} \approx 1.35$ for LiH to $\hat{R}_{\text{HT}}^{(15)}/\hat{R}_{\text{TPB}} \approx 1.50$ for HCN corroborates the usefulness of our new method in the realm of electronic structure problems.

TABLE II. Estimated shot reduction $\hat{R}$ for different Pauli groupings of molecular Hamiltonians with n qubits and M Pauli operators. TPB and GC groupings are obtained with the SI-QWC and SI algorithm, respectively [4]. For HT groupings, diagonalization circuits are tailored to the hardware connectivity[a] of *ibmq_guadalupe* using two variants of Algorithm 1, which differ by the objective function: *size only* as in Eq. (14) and *with coefficients* as in Eq. (15). Here, we use the `restricted algebraic solver` from Tab. III in the main text in combination with an exhaustive search over all subgraphs. For N_2 and HCN, we speed up computations by exploiting cutoffs $c_{N2} = 5$ and $c_{HCN} = 3$ as defined in Eq. (27) of the main text.

name	n	M	$\hat{R}_{TPB}$	$\hat{R}_{HT}^{(14)}$	$\hat{R}_{HT}^{(15)}$	$\hat{R}_{GC}$
LiH	4	99	6.57	7.78	8.86	8.75
H_4	5	120	6.76	10.69	10.39	14.65
H_2O	9	753	12.20	18.60	19.87	25.15
N_2	12	1,210	18.85	29.48	31.35	60.64
HCN	15	3,771	16.91	24.41	25.44	67.89

[a] Digital feature: clicking on the name of any molecule redirects to a web-page showing the assumed connectivity graph [30].

Influence of the value function

In SM Sec. V, we proposed two objective functions for quantifying the value of a collection: (14) *the number of elements it contains*, and (15) *a function that also takes coefficients into account.* A priori, it is unclear which objective function will lead to a better result. In Tab. II, we see $\hat{R}_{HT}^{(14)} < \hat{R}_{HT}^{(15)}$ for all examples except for H_4, where $\hat{R}_{HT}^{(14)} \gtrsim \hat{R}_{HT}^{(15)}$ holds. Because of this observation, we find it more promising to use the value function defined in Eq. (15). Thus, this option is used throughout this work as a default.

Carefully balancing hyperparameters

For the molecules N_2 and HCN, we report in Tab. II the performance of the `restricted algebraic solver` in combination with all available circuit templates. How much does the quality of the HT Pauli grouping suffer due to the cutoff restriction? To answer this question, we apply the `exhaustive informed numerical solver` from Tab. III in the main text. From our discussion of Fig. 5 in the main text, we know that this solver produces near-optimal HT Pauli groupings (at the cost of an exponential runtime). We find $\hat{R}_{HT}^{num} \approx 35.33$ compared to $\hat{R}_{HT}^{alg} \approx 31.35$ for N_2 and $\hat{R}_{HT}^{num} \approx 32.50$ compared to $\hat{R}_{HT}^{alg} \approx 25.44$ for HCN. With the `exhaustive informed numerical solver`, the total runtime was four hours for N_2 and an entire week for HCN. Computing for HCN the Pauli grouping with $\hat{R}_{HT}^{alg} \approx 25.44$, on the other hand, required "only" thirty hours. All computations were carried out on an 18 core Intel Xeon CPU E5-2697 v4 @2.30GHz device.

TABLE III. Balancing the hyperparameters of Algorithm 1 for the example of a molecular Hamiltonian for HCN with $n = 15$ qubits. The choice for the cutoff and the considered number of randomly selected subgraphs (see methods of the main text) influences the obtained grouping of the $M = 3,771$ Pauli operators into N_{HT} jointly-measurable collections, the estimated shot reduction $\hat{R}_{HT}$, and the preprocessing time t. We tailor the diagonalization circuits to the hardware connectivity of *ibmq_guadalupe* for which $2^{15} = 32,768$ subgraphs exist. All computations were carried out on an 18 core Intel Xeon CPU E5-2697 v4 @2.30GHz device.

cutoff	subgraphs	N_{HT}	$\hat{R}_{HT}$	$\frac{\hat{R}_{HT}}{\hat{R}_{TPB}}$	t [h]
0	1,000	701	20.15	1.19	0.9
	5,000	637	22.25	1.32	2.0
	all	615	23.26	1.38	22
2	1,000	648	21.44	1.27	1.0
	10,000	584	24.05	1.42	4.9
3	1,000	607	22.91	1.35	1.3
	5,000	560	24.23	1.43	3.6
	all	532	25.44	1.50	30
**5**	1,000	534	24.52	1.45	5.1
	**5,000**	486	**26.50**	1.57	**14**
7	100	600	21.89	1.29	43

Luckily, it is possible to carefully balance the hyperparameters of the `restricted algebraic solver` to both bring down the total runtime and simultaneously improve the value of $\hat{R}_{HT}$. In Tab. III, we show the results for various choices of hyperparameters at the example of the HCN molecule. Strikingly, we find a HT Pauli grouping with $\hat{R}_{HT} \approx 26.50$ in only fourteen hours by selecting a cutoff of $c = 5$ and a random subset of $s = 5,000$ circuit templates.

When balancing the hyperparameters of the `restricted algebraic solver`, there are two competing effects that occur: if the number of subgraphs is too small, we neglect well-suited readout circuit templates, which leads to a suboptimal value of $\hat{R}$. On the other hand, if it is too large, we redundantly construct circuits (for multiple subgraph) for Pauli operators for which a good circuit has already been found, which leads to an increase of t without improving $\hat{R}$. Similarly, if the cutoff is too large, we redundantly construct circuits by computing multiple single-qubit Clifford layers (for every single subgraph for which this is possible), which also increases t without improving $\hat{R}$. Conversely, if the cutoff is too small, we skip valuable circuit templates; even if they are included in the list of considered subgraphs. The latter is not as prohibitive as one could naively believe (cf. Fig. 7 in SM Sec. XI): even for a cutoff of $c = 0$, we obtain HT Pauli groupings that notably outperform $\hat{R}_{TPB} \approx 16.91$, see Tab. III.

In conclusion, Algorithm 1 can greatly benefit from a suitable choice of its hyperparameters. The study of the optimal strategy for selecting hyperparameters is beyond the scope of this work and requires further

TABLE IV. Influence of frequent renewal of the random selection of subgraphs on $\hat{R}_{\mathrm{HT}}/\hat{R}_{\mathrm{TPB}}$ for the example of parity-encoded hydrogen chains with eight ($n = 14$ qubits) and ten ($n = 18$) hydrogen atoms at an interatomic distance of $d = 1.0$ Å. Assuming linearly-connected qubits, we construct *hardware-tailored* (HT) Pauli groupings in two different ways. **(normal)** original form of Algorithm 1. **(modified)** after a collection of jointly-HT-diagonalizable Pauli operators is assigned (line 21 in Algorithm 1), we replace `subgraphs` with a newly-sampled random list of the same size.

n	cutoff	subgraphs	$\hat{R}_{\mathrm{HT}}^{\mathrm{normal}}/\hat{R}_{\mathrm{TPB}}$	$\hat{R}_{\mathrm{HT}}^{\mathrm{modified}}/\hat{R}_{\mathrm{TPB}}$
14	3	300	**2.15**	2.06
	3	500	**2.21**	2.12
	3	1,000	**2.30**	2.20
	3	2,000	**2.35**	2.34
	5	300	2.15	**2.32**
	5	2,000	**2.44**	2.34
18	3	500	2.24	**2.30**
	5	300	2.23	**2.26**
	5	2,000	**2.41**	2.20

investigation.

No gain through updating subgraphs on the fly

Finally, we test yet another modification of Algorithm 1 based on the possibility of resampling the set of random subgraphs every time a set of jointly-HT-diagonalizable Pauli operators is assigned. Although in this way the diversity of circuit templates is increased, we do not observe clear benefits compared to the original method, see Tab. IV. Only if the number of subgraphs is very small, we sometimes obtain a small advantage of the modified redrawing method. In practice, however, it is impractical to restrict to a very small number of subgraphs as this can easily lead to sub-optimal HT Pauli groupings. Further investigation is needed to better understand why changing the selection of subgraphs during the computation often leads to slightly worse Pauli groupings in Tab. IV.

VIII. DETAILED INVESTIGATION OF PAULI GROUPINGS FOR RANDOM HAMILTONIANS

Here we thoroughly discuss Figs. 5 **b**,**e**, and **h** from the main text, in which the estimated shot reduction ratio $\hat{R}_{\mathrm{HT}}/\hat{R}_{\mathrm{TPB}}$ for random Hamiltonians is shown. For every choice of the numbers n and M of qubits and Pauli operators, one sample set containing 20 random Hamiltonians is created. Then, we apply the *Sorted Insertion* (SI) algorithm [4], the SI-QWC algorithm, and two variants of the HT Pauli grouping algorithm introduced in Sec. V. For the latter, we tailor the diagonalization circuits to a linear hardware connectivity. While the figure in the main text only shows the ratio $\hat{R}_{\mathrm{HT}}/\hat{R}_{\mathrm{TPB}}$, we depict the individual values of $\hat{R}$ in Fig. 4. As expected, we observe $\hat{R}_{\mathrm{GC}} > \hat{R}_{\mathrm{HT}} > \hat{R}_{\mathrm{TPB}}$ for every fixed value of n and M. Furthermore, $\hat{R}$ increases (decreases) with M (with n) if n (if M) is held constant. To understand the reason for this, recall that every Pauli grouping method for $O = \sum_{i=1}^{M} c_i P_i$ partitions the set $\mathcal{X} = \{P_1, \ldots, P_M\}$ into a disjoint union

$$\mathcal{X} = \bigcup_{i=1}^{N} \mathcal{X}_i \tag{26}$$

of subsets $\mathcal{X}_i = \{P_{i,1}, \ldots, P_{i,m_i}\}$. Hereby, all operators in a given subset $\mathcal{X}_i$ can be simultaneously diagonalized with a *general* (GC), *hardware-tailored* (HT), or single-qubit (TPB) *Clifford circuit*, respectively. We expect that more shots can be saved (bigger $\hat{R}$) if more operators are simultaneously measurable (bigger m_i). Note that this rule of thumb is only valid because presorting the operators in $\mathcal{X}$ by the magnitude of their coefficients ensures that pathological Pauli groupings with highly suboptimal values of $\hat{R}$ are avoided [4]. As we show in Fig. 5, the average size $\bar{m}_i = M/N$ of the subsets $\mathcal{X}_i$ indeed reproduces the behavior of $\hat{R}$ remarkably well.

The curves of $\bar{m}_i$ and, therefore, $\hat{R}$ can be explained by graph-theoretical means. Consider the two graphs $G_{\mathrm{GC}}^{\mathrm{not}}$ and $G_{\mathrm{QWC}}^{\mathrm{not}}$, whose vertex sets are both given by $\mathcal{X} = \{P_1, \ldots, P_M\}$. The difference between the two graphs is the set of edges. As the names suggest, two vertices $P_i, P_j \in \mathcal{X}$ are connected by an edge in $G_{\mathrm{GC}}^{\mathrm{not}}$ ($G_{\mathrm{QWC}}^{\mathrm{not}}$) if the operators P_i and P_j are not GC (QWC). In other words, P_i and P_j are disconnected if the set $\{P_i, P_j\}$ is GC (QWC). By definition, a set of operators is GC (QWC) if and only if the same is true for all of its subsets of size two. Therefore, the GC (QWC) subsets $\mathcal{X}_i \subset \mathcal{X}$ are given by the completely disconnected subsets of the vertex set of $G_{\mathrm{GC}}^{\mathrm{not}}$ ($G_{\mathrm{QWC}}^{\mathrm{not}}$), i.e., those which do not contain a single pair of vertices sharing an edge. Thus, a Pauli grouping as in Eq. (26) is what in graph theory is known as a *coloring*: *a color is assigned to every vertex in a way that neighboring vertices have different colors.* It has been pointed out before that finding a coloring of $G_{\mathrm{GC}}^{\mathrm{not}}$ (of $G_{\mathrm{QWC}}^{\mathrm{not}}$) yields a Pauli grouping of $\mathcal{X}$ into subsets $\mathcal{X}_i$ that can be simultaneously diagonalized with general (with single-qubit) Clifford circuits [5, 19–21]. Unfortunately, this approach cannot be easily generalized to partition $\mathcal{X}$ into subsets $\mathcal{X}_i$ that can be simultaneously diagonalized with HT circuits. Consider, for example, the $M = 3$ operators $P_1 = XXZI$, $P_2 = YXYY$, and $P_3 = ZZZZ$ on $n = 4$ qubits whose hardware connectivity is given by the adjacency matrix

$$\Gamma = \begin{bmatrix} 0 & 1 & 1 & 1 \\ 1 & 0 & 0 & 0 \\ 1 & 0 & 0 & 0 \\ 1 & 0 & 0 & 0 \end{bmatrix}. \tag{27}$$

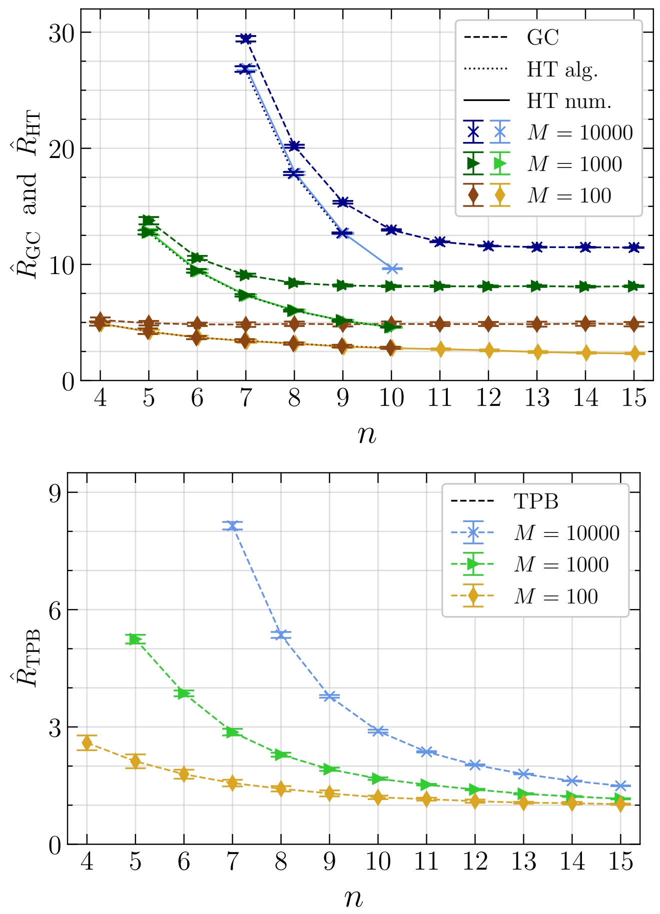

FIG. 4. Estimated shot reduction $\hat{R}$ for various Pauli groupings of the same random Hamiltonians as in the central panel of Fig. 5 in the main text as a function of the number n of qubits. Colors and markers correspond to the number M of Pauli operators, while shade and line style indicate the utilized solver. **(top)** GC (dark, dashed), HT alg. (dark, dotted), and HT num. (bright, solid). **(bottom)** TPB (bright, dashed).

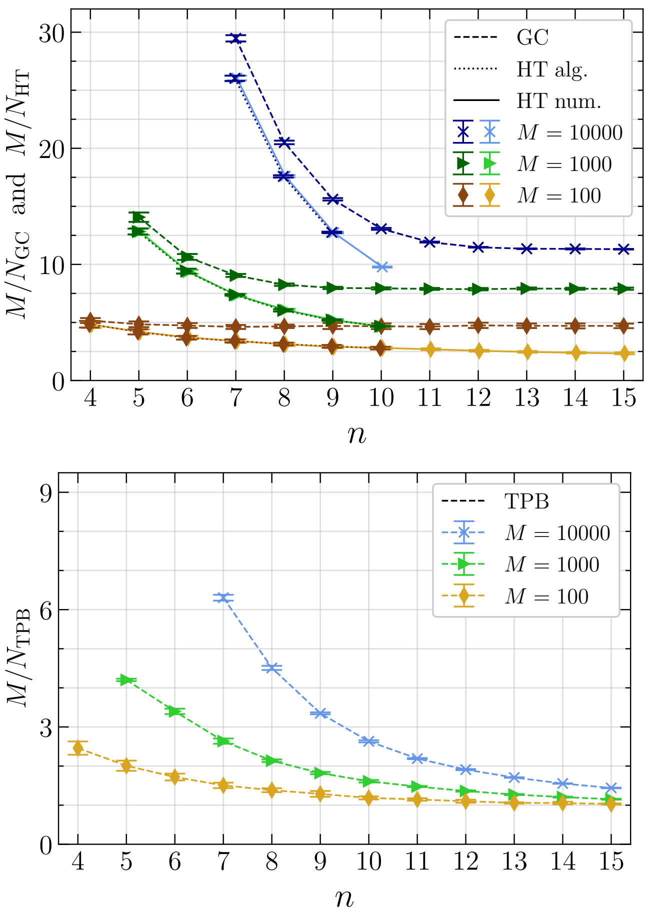

FIG. 5. Average number $\bar{m}_i = M/N$ of commuting Pauli operators $P_{i,1}, \ldots, P_{i,m_i}$ contained in $\mathcal{X}_i$, see Eq. (26), for various Pauli groupings of the same random Hamiltonians as in Fig. 4 as a function of the number n of qubits. Colors and markers correspond to the number M of Pauli operators, while shade and line style indicate the utilized solver. **(top)** GC (dark, dashed), HT alg. (dark, dotted), and HT num. (bright, solid). **(bottom)** TPB (bright, dashed).

For the sets $\{P_1, P_2\}$, $\{P_1, P_3\}$, and $\{P_2, P_3\}$, there exist HT diagonalization circuits but not for $\{P_1, P_2, P_3\}$. Because of obstructions like this, we focus on explaining the GC- and TPB-curves in Fig. 5 and are satisfied with pointing out that, intuitively, the HT-curves should lie between them, which is indeed the case. The working principle of all three here-considered Pauli grouping algorithms is to grow the subsets $\mathcal{X}_i$ by adding one Pauli operator at a time until no further operators can be added. In this way, the number N of resulting subsets is kept so small that it can approximately attain the *chromatic number* $\chi(G)$, which is defined as the minimal number of colors needed in a coloring of a graph G. This yields

$$\bar{m}_i = \frac{M}{N} \approx \frac{M}{\chi(G)}, \tag{28}$$

where $G = G^{\mathrm{not}}_{\mathrm{GC}}$ or $G = G^{\mathrm{not}}_{\mathrm{QWC}}$, depending on the problem. For every random Hamiltonian $O = \sum_{i=1}^{M} c_i P_i$, the vertex set $\mathcal{X} = \{P_1, \ldots, P_M\}$ of G is created by drawing M operators from $\{I, X, Y, Z\}^{\otimes n}$ at random without replacement. Within the limit of large qubit numbers $n \gg \log_4(M)$, it is irrelevant that the operators are picked without replacement, and it is justifiable to assume that $G^{\mathrm{not}}_{\mathrm{GC}}$ ($G^{\mathrm{not}}_{\mathrm{QWC}}$) is an Erdős-Rényi random graph with M vertices and a vertex-independent edge probability of $\frac{1}{2}$ (of approximately 1) because the probability that two random Pauli operators commute (on every qubit) is given by $\frac{1}{2}$ (is exponentially suppressed in n) [20]. In particular, $\chi(G^{\mathrm{not}}_{\mathrm{QWC}})$ converges to the chromatic number $\chi(K_M) = M$ of the *complete graph* K_M with M vertices. Therefore, Eq. (28) yields

$$\lim_{n\to\infty} \bar{m}_{i,\mathrm{TPB}} \approx 1, \tag{29}$$

which implies $\hat{R}_{\text{TPB}} \to 1$ and explains the asymptotic behavior of the curves in the lower panels of Fig. 5 and Fig. 4. Likewise, $\chi(G_{\text{GC}}^{\text{not}})$ converges to the chromatic number of an M-vertex, $\frac{1}{2}$-edge-probability Erdős-Rényi random graph G_{ran}, which (with high probability) is given by $\chi(G_{\text{ran}}) = M/(\gamma(M) + o(1))$, where

$$\gamma(M) = 2\log_2(M) - 2\log_2(\log_2(M)) - 2\log_2(2) \quad (30)$$

was introduced in Ref. [31]. Therefore, we expect that the asymptotes

$$\lim_{n\to\infty} \bar{m}_{i,\text{GC}} \approx \gamma(M) + o(1) \quad (31)$$

are logarithmically growing in the number M of Pauli operators. Now that we understand its asymptotic behavior, let us discuss why $\bar{m}_i$ increases when n is *decreased* while M is held constant. The key difference between G_{ran} and $G_{\text{GC}}^{\text{not}}$ (K_M and $G_{\text{QWC}}^{\text{not}}$) is that G_{ran} can have (K_M only has) vertices $P \in \mathcal{X}$ whose *degree*

$$\deg_G(P) = |\{P' \in \mathcal{X} \mid P, P' \text{ share an edge in } G\}| \quad (32)$$

reaches the trivial bound $\deg_G(P) \leq M - 1$, whereas for $G_{\text{GC}}^{\text{not}}$ ($G_{\text{QWC}}^{\text{not}}$) it cannot exceed the total number of operators $P' \in \{I, X, Y, Z\}^{\otimes n}$ that do not (qubitwise) commute with P. In other words,

$$\deg_G(P) \leq \begin{cases} 4^n/2 & \text{if } G = G_{\text{GC}}^{\text{not}} \\ 4^n - 2^k 4^{n-k} & \text{if } G = G_{\text{QWC}}^{\text{not}}, \end{cases} \quad (33)$$

where $k = \text{wt}(P)$ is the *Pauli weight* of P, i.e., its number of non-identity tensor factors. In combination with Brooks' theorem,

$$\chi(G) \leq 1 + \max_{P\in\mathcal{X}} \deg_G(P), \quad (34)$$

Ineq. (33) causes the chromatic number of $G_{\text{GC}}^{\text{not}}$ ($G_{\text{QWC}}^{\text{not}}$) to shrink when n is decreased. From Eq. (28), this in turn gives room for $\bar{m}_i$ to grow, which is indeed what we observe in Fig. 5. Finally, let us explain the dependence of $\bar{m}_i$ on M for fixed n. Assume we have a Pauli grouping as in Eq. (26) and we start to reduce the value of M by randomly removing some operators from $\mathcal{X}$. It is more likely that these operators are removed from different subsets $\mathcal{X}_i$ (which decreases m_i) than it is that one of the subsets is emptied completely (which would decrease $N = M/\bar{m}_i$). This is why $\bar{m}_i$ increases with M in Fig. 5. For example, the average sizes of GC subsets on $n = 7$ qubits are given by $\bar{m}_i(M = 10^2) \approx 5.2$, $\bar{m}_i(M = 10^3) \approx 14.1$, and $\bar{m}_i(M = 10^4) \approx 29.5$. Although our graph-theoretical arguments break down in the case of HT Pauli groupings, we see in Fig. 4 that, qualitatively, $\hat{R}_{\text{HT}}$ behaves like $\hat{R}_{\text{GC}}$ and $\hat{R}_{\text{TPB}}$.

IX. HAMILTONIAN EXPONENTIATION

In this section, we address a fundamental component of quantum computation that could potentially benefit from *hardware-tailored* (HT) Pauli groupings: decompositions of unitary gates. The phase gate $\exp(icZ)$ with $c \in \mathbb{R}$, for example, is a basic single-qubit gate, which can be directly implemented on most quantum hardware [32]. Similarly, $\textsc{Cnot} = |0\rangle\langle 0| \otimes I + |1\rangle\langle 1| \otimes X$ is a basic two-qubit gate that flips the state of the second (target) qubit, depending on the state of the first (control) qubit. It is well known that every unitary gate is of the form $\exp(iO)$ for some Hermitian operator O [33]. Furthermore, $\exp(iO)$ can be decomposed into a sequence of Cnot gates, Hadamard gates $H = \frac{1}{\sqrt{2}}(X + Z)$, and phase gates $\exp(icZ)$ with $c \in \mathbb{R}$, e.g., $\exp(icZ \otimes Z) = \textsc{Cnot}(I \otimes \exp(icZ))\textsc{Cnot}$. Likewise, the n-qubit unitary

$$\exp(icZ^{\mathbf{s}}) = \left(\prod_{j=1}^{n-1} \textsc{Cnot}_{j,n}^{s_j}\right)\left(I^{\otimes(n-1)} \otimes \exp(icZ)\right)\left(\prod_{j=1}^{n-1} \textsc{Cnot}_{j,n}^{s_j}\right), \quad (35)$$

can be implemented with the quantum circuit depicted in Fig. 6, where the binary vector $\mathbf{s} \in \mathbb{F}_2^n$ determines the involved qubits, and we assumed $s_n = 1$ to ease notation. Next, let $P \in \{I, X, Y, Z\}^{\otimes n}$ be a Pauli operator and U a quantum circuit diagonalizing it, i.e., $UPU^\dagger = \pm Z^{\mathbf{s}}$ for some $\mathbf{s} \in \mathbb{F}_2^n$ (see main text, Fig. 1). Then, an expansion of the exponential series yields

$$\exp(icP) = U^\dagger \exp(\pm icZ^{\mathbf{s}})U. \quad (36)$$

Hence, the unitary $\exp(icP)$ can be implemented by first applying U, then $\exp(\pm icZ^{\mathbf{s}})$, then $U^\dagger$.

Finally, consider the most general case in which O is a sum of Pauli operators $P_i \in \{I, X, Y, Z\}^{\otimes n}$ with coefficients $c_i \in \mathbb{R}$. As thoroughly discussed in SM Sec. V, applying a Pauli grouping algorithm decomposes O into

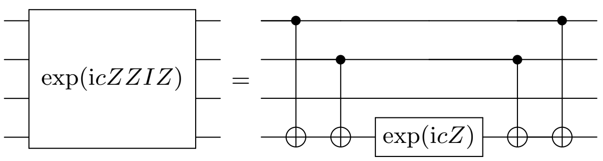

FIG. 6. Example of the circuit identity in Eq. (35) for $n = 4$ qubits and $\mathbf{s} = [1, 1, 0, 1]^{\mathrm{T}}$. Note that the outer CNOT gates could alternatively target qubit 2 instead of qubit 4, which would require fewer SWAP gates on a chip with linearly connected qubits.

a sum $O = \sum_{i=1}^{N} O_i$ of fragment operators

$$O_i = \sum_{j=1}^{m_i} c_{i,j} P_{i,j}. \tag{37}$$

By construction, the Pauli operators $P_{i,1}, \ldots, P_{i,m_i}$ can be simultaneously diagonalized with a Clifford circuit U_i, i.e., there exist binary vectors $\mathbf{s}^{(i,j)}$ such that

$$U_i P_{i,j} U_i^\dagger = \pm Z^{\mathbf{s}^{(i,j)}} \tag{38}$$

for every $i \in \{1, \ldots, N\}$ and $j \in \{1, \ldots, m_i\}$. In particular, the operators $P_{i,1}, \ldots, P_{i,m_i}$ commute, which yields the decomposition

$$\exp(\mathrm{i}O_i) = \prod_{j=1}^{m_i} \exp(\mathrm{i}c_{i,j} P_{i,j}). \tag{39}$$

By inserting Eqs. (38)–(37) into Eq. (39) and cancelling basis-change circuits of the form $U_i^\dagger U_i$, we find

$$\exp(\mathrm{i}O_i) = U_i^\dagger \left(\prod_{j=1}^{m_i} \exp\left(\pm \mathrm{i} c_{i,j} Z^{\mathbf{s}^{(i,j)}} \right) \right) U_i. \tag{40}$$

We point out that by carefully selecting the ordering of the commuting gates $\exp(\pm \mathrm{i} c_{i,j} Z^{\mathbf{s}^{(i,j)}})$, it might be possible to cancel some of the CNOT gates stemming from Eq. (35). In order to finally decompose the gate $\exp(\mathrm{i}O)$, one has to be careful because, in general, the operators $O_1, \ldots, O_N$ do not commute. In principle, $\exp(\mathrm{i}O)$ can be exactly implemented with an infinitely long quantum circuit

$$\exp(\mathrm{i}O) = \lim_{k \to \infty} \left(\prod_{i=1}^{N} \exp\left(\tfrac{\mathrm{i}O_i}{k} \right) \right)^k, \tag{41}$$

known as the Lie-Trotter product formula [34]. In practice, however, Eq. (41) is truncated at a finite value of k, which yields the approximation

$$\exp(\mathrm{i}O) \approx \left(\prod_{i=1}^{N} U_i^\dagger \left(\prod_{j=1}^{m_i} \exp\left(\pm \mathrm{i} \tfrac{c_{i,j}}{k} Z^{\mathbf{s}^{(i,j)}} \right) \right) U_i \right)^k. \tag{42}$$

In conclusion, the final circuit in Eq. (42) contains $2Nk$ subcircuits for diagonalization (U_i and $U_i^\dagger$) as well as Mk rotations of the form $\exp(\mathrm{i}cZ^{\mathbf{s}})$.

For applications in quantum chemistry, where O is the Hamiltonian of a molecule, the number M of Pauli operators scales with the fourth power of number n of qubits [18]. The cost of the diagonalization circuits and their number N depends on the Pauli grouping algorithm in place. If the Pauli operators are grouped into tensor product bases, every U_i is just a layer of single-qubit Clifford gates but, typically, there are as many as $N_{\mathrm{TPB}} \approx M/3 = \mathcal{O}(n^4)$ of them [19]. On the other extreme, empirically [5, 20], only $N_{\mathrm{GC}} = \mathcal{O}(n^3)$ diagonalization circuits are needed if the Pauli operators are grouped into general commuting sets. In this case, however, the diagonalization circuits U_i require up to $n(n-1)/2$ two-qubit gates and an additional number of SWAP gates [35]. Finally, for HT Pauli groupings, the diagonalization circuits have a constant depth and are tailored to the hardware connectivity. We leave the question of which connectivities are needed such that N_{HT} scales better than $\mathcal{O}(n^4)$ as an open problem.

In every iteration of our Pauli grouping algorithm (Algorithm 1 in Sec. V), multiple diagonalization circuits are created and only the "best" one is kept. If the purpose of the constructed Pauli grouping is to speed up estimating $\langle O \rangle$, "best" is heuristically quantified by the number of jointly-measurable Pauli operators (weighted with coefficients), see Eq. (15) in Sec. V. For Hamiltonian exponentiation, on the other hand, its purpose is a resource-efficient implementation of the Trotter-step circuit in Eq. (42) and the above heuristic is likely suboptimal: even though such a Pauli grouping would approximately minimize the number N of basis-change circuit components $U_{i+1} U_i^\dagger$ in Eq. (42), the problem of resource-efficiently implementing $\exp(\pm \mathrm{i} \frac{c_{i,j}}{k} Z^{\mathbf{s}^{(i,j)}})$ would remain unaddressed. Since we need to implement Mk circuits of the form $\exp(\mathrm{i}cZ^{\mathbf{s}})$ but only $Nk < Mk$ basis-change circuits, implementing the former is the true bottleneck. Note that the support of the exponent vectors $\mathbf{s}^{(i,j)}$ influences the number of CNOT and SWAP gates in the trees of CNOT gates that are needed for the implementation of $\exp(\pm \mathrm{i} \frac{c_{i,j}}{k} Z^{\mathbf{s}^{(i,j)}})$ as in Fig. 6. Algorithm 1 is flexible and would allow us to incorporate such information as we could replace the notion of "best" from Eq. (15) by a figure of merit that punishes choices of U_i for which $\mathbf{s}^{(i,1)}, \ldots, \mathbf{s}^{(i,m_i)}$ leads to a large overhead in SWAP gates. We are optimistic that such an approach would enable access to resource-efficient HT Hamiltonian exponentiation and encourage the community to explore this opportunity in future research.

X. HOW TO COMPUTE INTERSECTIONS OF AFFINE SUBSPACES

Let us explain in greater detail how the intersection $\mathcal{L}_i = \mathcal{W}_i^{(1)} \cap \mathcal{Y}_i^{(1)}$ from Eq. (21) of the main text is treated computationally. We can assume that neither $\mathbf{w}_i$ nor $\mathbf{y}_i$ are equal to zero because otherwise we would be in the trivial case of $\mathcal{L}_i = \varnothing$. Hence, the linear map $\mathbb{F}_2^d \to \mathbb{F}_2$, $\boldsymbol{\lambda} \mapsto \boldsymbol{\lambda}^{\mathrm{T}}\mathbf{w}_i$ is surjective, and, by the rank-nullity theorem, its null space $\mathcal{W}_i^{(0)}$ has dimension $d-1$. Let $j_1 \in \{1, \ldots, d\}$ denote a position for which $w_{i,j_1} = 1$. For every other index $j \neq j_1$, we obtain a basis vector for $\mathcal{W}_i^{(0)}$, which stores the value 1 in position j, the value of $w_{i,j}$ in position j_1, and 0 in all other positions. The elements of $\mathcal{W}_i^{(0)}$ and $\mathcal{W}_i^{(1)}$ are in one-to-one correspondence to each other via the addition of a support vector $\mathbf{w}_i' \in \mathcal{W}_i^{(1)}$. For the latter, we can pick the basis vector $\mathbf{e}_{j_1} = [0, \ldots, 0, 1, 0, \ldots, 0]^{\mathrm{T}} \in \mathbb{F}_2^d$, where the 1 is in position j_1. Analogously, we can find a basis and a support vector for $\mathcal{Y}_i^{(1)}$. As we show next, the intersection $\mathcal{L}_i$ of the two affine subspaces $\mathcal{W}_i^{(1)}$ and $\mathcal{Y}_i^{(1)}$ is indeed an affine subspace.

For pedagogical reasons, we now explain how to computationally carry out the intersection $\mathcal{U} \cap \tilde{\mathcal{U}}$ of two affine subspaces $\mathcal{U}, \tilde{\mathcal{U}} \subset \mathbb{K}^d$, where $\mathbb{K}$ can be any field. Let $\mathbf{u}_1, \ldots, \mathbf{u}_m$, and $\tilde{\mathbf{u}}_1, \ldots, \tilde{\mathbf{u}}_{\tilde{m}}$ be bases of $\mathcal{U}$ and $\tilde{\mathcal{U}}$, respectively. Furthermore, pick $\mathbf{u}' \in \mathcal{U}$ and $\tilde{\mathbf{u}}' \in \tilde{\mathcal{U}}$. This yields a parametrization $\mathcal{U} = \{\mathbf{u}' + \sum_{i=1}^m \mu_i \mathbf{u}_i \mid \mu_i \in \mathbb{K}\}$ and similarly for $\tilde{\mathcal{U}}$. To compute a basis and a support vector of $\mathcal{U} \cap \tilde{\mathcal{U}}$, we define two matrices $U \in \mathbb{K}^{d \times m}$, $\tilde{U} \in \mathbb{K}^{d \times \tilde{m}}$, whose columns are given by the basis vectors of $\mathcal{U}$ and $\tilde{\mathcal{U}}$, respectively. Via Gaussian elimination over $\mathbb{K}$, we can bring the matrix $U' = [U, -\tilde{U}, \mathbf{u}' - \tilde{\mathbf{u}}'] \in \mathbb{K}^{d \times (m+\tilde{m}+1)}$ to its *reduced row-echelon form* (RREF) and read off a basis $\mathbf{t}_1, \ldots \mathbf{t}_\ell \in \mathbb{K}^{m+\tilde{m}}$ of the null space of $[U, -\tilde{U}]$. If we denote the truncation of these basis vectors to the first m rows by $\bar{\mathbf{t}}_i = [t_{i,1}, \ldots, t_{i,m}]^{\mathrm{T}}$ for all $i \in \{1, \ldots, \ell\}$, a basis of $\mathcal{U} \cap \tilde{\mathcal{U}}$ is given by $U\bar{\mathbf{t}}_1, \ldots, U\bar{\mathbf{t}}_\ell \in \mathbb{K}^d$. Finally, if the last column of U' is a non-pivot column, i.e., $\mathrm{rank}(U') = \mathrm{rank}([U, -\tilde{U}])$, then the last column of the RREF of U' is a support vector for $\mathcal{U} \cap \tilde{\mathcal{U}}$. Otherwise, $\mathcal{U} \cap \tilde{\mathcal{U}}$ is empty.

XI. DEPENDENCE OF HT-IDENTIFIABILITY ON THE CUTOFF

Here we study how the choice of the cutoff c, which is introduced in Eqs. (27)–(28) of the main text, influences the performance of the `restricted algebraic solver` from Tab. III of the main text. To better understand how much information is lost when the cutoff is applied, we now consider a quantum device with $n = 5$ linearly connected qubits such as the superconducting quantum computer *ibmq_santiago*. The associated connectivity graph possesses four edges and 2^4 subgraphs.

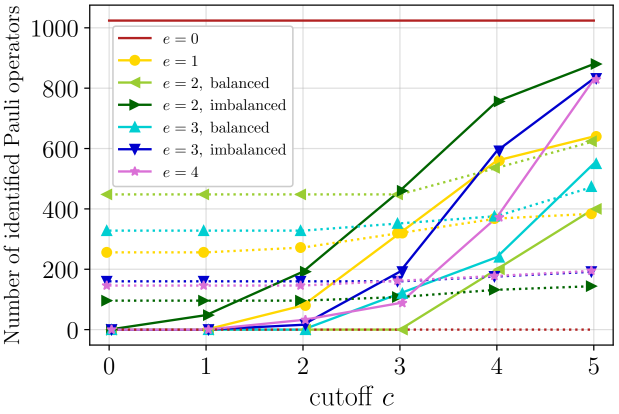

FIG. 7. Number of Pauli operators that are identified as diagonalizable (solid lines) or non-diagonalizable (dotted lines) by a graph-based circuit as in Fig. 1 of the main text for the 6 subgraph classes of a 5-vertex path graph as a function of the cutoff c. Here, each circuit only needs to diagonalize a single operator. The colors and markers indicate the number e of the subgraphs' edges and, for the ambiguous cases, whether the distribution of edges among the graph components is balanced or not. For the subgraph with $e = 0$ edges (red, no markers), we did not apply the cutoff because individual Pauli operators can be trivially diagonalized by single-qubit Clifford gates.

Every subgraph corresponds to a template that can be complemented with a layer of single-qubit Clifford gates to a HT circuit (see Fig. 1 of the main text). For every subgraph, we investigate how many of the $4^n = 1024$ Pauli operators can be **individually** diagonalized with such a circuit. If we succeed in finding a solution $\boldsymbol{\lambda} \in \mathcal{L}$ for a given cutoff c, the corresponding Pauli operator P is identified as being *diagonalizable*.

In Fig. 7, we plot the number of Pauli operators identified as diagonalizable as a function of c (solid lines). Hereby, different lines correspond to different subgraphs. Furthermore, we plot the number of operators P that can be identified as *non-diagonalizable* (dotted lines), i.e., the cases where $\mathcal{L}_1 \cap \ldots \cap \mathcal{L}_{n-k} = \varnothing$ or where $Q_i = 0$ for one of the qubits i. Hereby, k again denotes the number of rank-2 hypersurfaces $\mathcal{L}_i$. For completeness, we include the trivial subgraph with $e = 0$ edges for which every Pauli operator can be individually diagonalized with the standard, qubitwise diagonalization circuit (H for X, $HS^\dagger$ for Y, and I for I and Z). For the other cases (with $e > 0$ edges), we consistently observe the following behavior: for the most drastic cutoff of $c = 0$, a considerable portion of the set of non-diagonalizable operators is recognized, whereas not a single diagonalizable operator can be identified. As expected, the number of identifiable Pauli operators monotonically increases when the cutoff c is enlarged. Eventually, when no cutoff is applied ($c = n$), we are able to classify the entire set of Pauli operators.

Let us now compare curves corresponding to different

subgraphs. Since the considered problem is symmetric under graph isomorphisms, the (yellow) curves for all graphs with $e = 1$ coincide. For subgraphs with $e = 2$ (green) and $e = 3$ (blue), respectively, there are two classes we call *balanced* (①–② ③–④ ⑤ for $e = 2$ and ①–②–③ ④–⑤ for $e = 3$) and *imbalanced* (①–②–③ ④ ⑤ for $e = 2$ and ①–②–③–④ ⑤ for $e = 3$), referring to the sizes of the subgraphs' connected components. For every choice of $c > 0$, the largest number of Pauli operators can be diagonalized with the HT circuit corresponding to an imbalanced subgraph with $e = 2$ edges (dark green), whereas the fewest number of Pauli operators is diagonalizable with circuits based on a balanced subgraph with $e = 2$ (bright green). A similar but less pronounced behavior is observed for the templates with $e = 3$ edges. As we are going to explain below, this is due to the existence of lone edges, i.e., connected components of the subgraph of size two.

Finally, note that, except for ①–② ③–④ ⑤, every subgraph allows us to diagonalize the majority of the Pauli operators. This abundance of HT-diagonalizable operators partially explains why the `restricted algebraic solver` is able to produce HT Pauli groupings with $\hat{R}_{\mathrm{HT}}/\hat{R}_{\mathrm{TPB}} > 1$, see Fig. 5 in the main text.

To understand why lone edges in a given subgraph lead to a large number of non-diagonalizable Pauli operators, we first explain the simpler case of two-qubit HT circuits that feature exactly one CZ gate. If followed by a qubitwise readout, such a circuit U realizes a Bell measurement, which is a crucial building block of the well-known quantum teleportation protocol [36]. Note that U can only rotate an entangled basis to the computational basis. In other words, if U transforms P_1, P_2, and P_3 into $I \otimes Z$, $Z \otimes I$, and $Z \otimes Z$, then every P_i must have a Pauli weight of two, i.e., $P_i \in \{X, Y, Z\}^{\otimes 2}$ up to a global phase. In summary, among all $4^2 = 16$ two-qubit Pauli operators the six weight-one operators are not diagonalizable, while $I \otimes I$ as well as the nine weight-two operators are diagonalizable by a circuit based on the graph ①–②. Next, consider the balanced four-qubit graph ①–② ③–④. A Pauli operator of the form $P = P^{(1)} \otimes \ldots \otimes P^{(4)}$ is non-diagonalizable if and only if the same is true either for $P^{(1)} \otimes P^{(2)}$ and ①–②, for $P^{(3)} \otimes P^{(4)}$ and ③–④, or for both. If $P^{(1)} \otimes P^{(2)}$ is non-diagonalizable (6 cases), the choice of $P^{(3)} \otimes P^{(4)}$ is irrelevant (16 cases); if, however, $P^{(1)} \otimes P^{(2)}$ is diagonalizable (10 cases), $P^{(3)} \otimes P^{(4)}$ has to be non-diagonalizable (6 cases). This gives a total amount of $6 \times 16 + 10 \times 6 = 156$ non-diagonalizable Pauli operators for the graph ①–② ③–④. It follows that there exist exactly $156 \times 4 = 624$ non-diagonalizable Pauli operators for the graph ①–② ③–④ ⑤ because the choice of the fifth operator is irrelevant. This explains why the bright green, dotted curve ($e = 2$, balanced) in Fig. 7 takes the comparatively large (i.e., bad) value of 624 for $c = 5$.

More generally, we can state that a graph will have a large amount of non-diagonalizable Pauli operators if it possesses a lot of two-vertex components (lone edges). Even more generally, we expect a drop in diagonalizable operators for every component with the property *every vertex has an odd number of neighbors* as this will make it impossible to diagonalize odd-weight Pauli operators on the subset of qubits that belong to such a component [37, 38].

XII. BREAKING THE EXPONENTIAL RUNTIME FOR FACTORIZABLE CIRCUITS

For subgraphs Γ with multiple connected components, we can enormously speed up finding a solution $\boldsymbol{\lambda} \in \mathcal{L}$ by breaking down the problem, individually solving it for each component, and finally combining the solutions of the subproblems. Indeed, if $\Gamma = \Gamma_1 \oplus \Gamma_2$ is the direct sum of two adjacency matrices Γ_1 and Γ_2 with n_1 and $n_2 = n - n_1$ qubits, respectively, the left-hand side of Eq. (6) in the main text becomes

$$\begin{bmatrix}\Gamma_1 & \\ & \Gamma_2\end{bmatrix}\left(\begin{bmatrix}A_1^{xx} & \\ & A_2^{xx}\end{bmatrix}\begin{bmatrix}R_1\\ R_2\end{bmatrix}+\begin{bmatrix}A_1^{xz} & \\ & A_2^{xz}\end{bmatrix}\begin{bmatrix}S_1\\ S_2\end{bmatrix}\right), \tag{43}$$

where we have defined $A_1^{xx} = \mathrm{diag}(a_1^{xx}, \ldots, a_{n_1}^{xx})$, $A_2^{xx} = \mathrm{diag}(a_{n_1+1}^{xx}, \ldots, a_n^{xx})$, and similarly for A^{xz}. Furthermore, R_1 and R_2 are given by the first n_1 and last n_2 rows of R, respectively, and likewise for S. By multiplying out expression (43) in a block-wise manner, we find that Eq. (6) of the main text is equivalent to

$$\begin{bmatrix}\Gamma_1(A_1^{xx}R_1 + A_1^{xz}S_1)\\ \Gamma_2(A_2^{xx}R_2 + A_2^{xz}S_2)\end{bmatrix} = \begin{bmatrix}A_1^{zx}R_1 + A_1^{zz}S_1\\ A_2^{zx}R_2 + A_2^{zz}S_2\end{bmatrix}. \tag{44}$$

While the original problem had a worst-case scaling that was exponential in n, solving the two decoupled equations in Eq. (44) has a worst-case scaling of $\tilde{\mathcal{O}}(\exp(n_1) + \exp(n_2))$. We emphasize that this breaking of the exponential scaling guarantees the efficient construction (if they exist) of single-qubit Clifford layers for graphs for which the size of the largest connected component grows at most logarithmically in n.

XIII. DETAILS ABOUT THE EXPERIMENT

In Fig. 3 of the main text, we report on an experiment in which hardware-tailored (HT) readout circuits outperform conventional *tensor product bases* (TPBs) measurements. Here, we provide more background information about the experiment.

Choice of Hamiltonian and TPB grouping

We start by constructing an eight-qubit molecular Hamiltonian O representing four hydrogen atoms

TABLE V. Pauli grouping of the H_4-Hamiltonian from Tab. VI into nine general commuting (GC) collections. The first Pauli operator and the total number of operators in collection i are denoted by $P_{i,1}$ and m_i, respectively. For every GC collection, we compute a readout circuit (see Fig. 8 for an example) using the algorithm provided in Ref. [5] in combination with SM Sec. III. Then, we use Qiskit to transpile the GC circuits to a linear connectivity by introducing SWAP gates [29]. The final number of two-qubit gates and SWAP gates in the transpiled circuits is presented.

i	$P_{i,1}$	m_i	#CNOT + #CPHASE	#SWAP
1	ZZZIZIII	36	0	0
2	IXIIIXII	24	0	0
3	IXXIIXXI	20	39	66
4	IYYXIYYX	24	32	54
5	IIXIIIII	16	27	45
6	IXXIIXII	16	34	59
7	IXIIIXXI	16	31	55
8	ZIXZIZZI	16	45	78
9	IYYXZZZI	16	34	54

on a linear chain with equal interatomic distances of $d = 1.0\,\text{Å}$. Then, we harness the SI-QWC algorithm (see SM Sec. V) to group the $M = 184$ operators in the Pauli decomposition of O into $N_{\text{TPB}} = 35$ TPBs and show the corresponding result in Tab. VI. As expected, the number of operators in the different TPBs is far from uniformly distributed [5, 20, 23]. This is undesirable because it leads to suboptimal shot reductions. The uneven distribution is due to Pauli operators with a low weight (number of non-identity tensor factors) running out first during the execution of the SI-QWC algorithm, which results in increasingly smaller TPBs, e.g., the last five TPBs only contain a single Pauli operator. For larger Hamiltonians, these effects are much more severe, see Fig. 2 in SM Sec. VI for an example.

GC grouping of the Hamiltonian

In theory, *general commuting* (GC) Pauli groupings would improve the situation. Applying the *Sorted Insertion* (SI) algorithm [4] to O results in only $N_{\text{GC}} = 9$ GC collections (see Tab. V). Note that GC collection 1 and 2 coincide with TPB 1 ($Z^{\otimes 8}$) and 2 [$(Z \otimes X)^{\otimes 4}$] from Tab. VI. The other GC collections, however, contain pairs of Pauli operators that are not qubitwise commuting. Their corresponding GC readout circuits contain a substantial amount of two-qubit gates and require a large SWAP gate overhead on a linear hardware connectivity (see Tab. V). As an illustrative example, we depict the readout circuit of the third GC collection in Fig. 8.

HT grouping of the Hamiltonian

We also compute a HT Pauli grouping by applying Algorithm 1 from SM Sec. V (see Tab. VII for the resulting HT collections and their diagonalization circuits). The result is impressive: compared to TPBs, the number of required readout circuits is reduced by a factor of 3.5, at a moderate increase in the average number of CZ gates from zero to two. Compared to GC circuits, the average number of two-qubit gates is reduced by a factor of 13 and, in addition, we avoid all 411 SWAP gates, at a moderate increase from $N_{\text{GC}} = 9$ to $N_{\text{HT}} = 10$. Thus, even for trapped-ion and neutral-atom quantum computers which feature all-to-all connectivity our HT Pauli groupings offer advantages over GC Pauli groupings.

Experimental procedure

Now, we explain how we have gathered the experimental data in Fig. 3 of the main text. All experiments were performed using eight linearly connected qubits on the superconducting quantum processor *imbq_washington*, see Tab. X for its device specifications during the experiment. The experimental procedure is as follows: First, we initialize each of qubits in a random state by applying a gate of the form

$$U_3(\theta, \phi, \lambda) = \begin{pmatrix} \cos\left(\frac{\theta}{2}\right) & -e^{i\lambda}\sin\left(\frac{\theta}{2}\right) \\ e^{i\phi}\sin\left(\frac{\theta}{2}\right) & e^{i(\phi+\lambda)}\cos\left(\frac{\theta}{2}\right) \end{pmatrix}, \qquad (45)$$

see Tab. VIII for our choice of the parameters θ_j, ϕ_j, and λ_j. Then, we execute one of the diagonalization circuits. Finally, we read out all qubits in the computational basis. For each diagonalization circuit (35 TPBs and 9 HT), we collect the measurement data from 10 million (10M) circuit executions. After applying readout error mitigation [2], the prevalent errors in the processed measurement results are likely due to imperfect gate operations in the diagonalization circuits. For a given shot budget N^{shots}, we compute the error $\epsilon = |E_{\text{exp}}(N^{\text{shots}}) - E_{\text{theo}}|$, where $E = \langle\psi| O |\psi\rangle$ is the energy of the ideal state. Hereby, we split a total amount of 50M shots into $\lfloor 50\text{M}/N^{\text{shots}} \rfloor$ subsets, where the ratios in Tab. IX are obeyed. For small values of N^{shot}, the number of repetitions is so large that the error on the mean of ϵ becomes negligible. The final results are shown in Fig. 3 of the main text.

Correct interpretation of the measurement data

Finally, let us point out an important technical detail. Any diagonalization circuit for a list of Pauli operators $P_1, \ldots, P_m \in \{I, X, Y, Z\}^{\otimes n}$ brings each P_i into the form $\pm Z^{\mathbf{k}_i}$ for some $\mathbf{k}_i \in \mathbb{F}_2^n$. For the correct interpretation of the measurement results it is

crucial to know the correct sign of $\pm Z^{\mathbf{k}_i}$; it can be reconstructed from $P_1, \dots, P_m$ via the prefactor map $\alpha : \mathbb{F}_2^{2n} \to \mathbb{Z}/4\mathbb{Z} = \{0,1,2,3\}$ for the layer of single-qubit Clifford gates represented by $A \in \mathrm{GL}(\mathbb{F}_2^{2n})$, which arises as

$$\alpha(\mathbf{r}, \mathbf{s}) = \sum_{j=1}^{n} \alpha_j(r_j, s_j) \tag{46}$$

from the single-qubit prefactor maps $\alpha_j : \mathbb{F}_2^2 \to \mathbb{Z}/4\mathbb{Z}$, represented by $A_j \in \mathrm{GL}(\mathbb{F}_2^2)$ (see main text, Tab. I). Note that $\alpha_j(1,1) = \alpha_j(1,0) + \alpha_j(0,1) + 2a_j^{xz}a_j^{zx}$. For the HT circuits used in this experiment, we provide the signs in Tab. VII.

XIV. BASIS SETS BEYOND STO-3G

Until this point, we have restricted our discussion of molecular Hamiltonians to the most simple case where orbitals are represented in the STO-3G basis. In this final supplementary section, we demonstrate that the advantage of our new *hardware-tailored* (HT) readout circuits over conventional *tensor product basis* (TPB) measurements can also be attained in the case of more complex basis sets beyond STO-3G.

For concreteness, we study the symmetric stretch of an H_2O molecule at an opening angle of $\alpha = 104.51°$ and various OH interatomic distances d. We consider two basis sets, 6-31G and 6-31G*, which yields Hamiltonians on $n = 26$ and $n = 38$, respectively. The final number M of Pauli operators in the molecular Hamiltonian shows small dependence on the OH distance, see Tab. XI.

First, we want to identify a meaningful hyperparameter choice for our Pauli grouping algorithm. For this, we concentrate on the chemically more challenging regime of large OH distances [41]. In the upper panel of Fig. 9, we show for $d = 2.552$ Å the estimated shot reduction $\hat{R}_{\mathrm{HT}}$ as defined in Ref. [4] as a function of the number s of random subgraphs used for the construction of the HT Pauli grouping. Moreover, we show $\hat{R}_{\mathrm{TPB}}$ as computed with Sorted Insertion [4], which serves as a baseline upon which $\hat{R}_{\mathrm{HT}}$ is supposed to improve. As expected, $\hat{R}_{\mathrm{HT}}$ grows with s and, starting from $s = 10^2$ subgraphs, we observe $\hat{R}_{\mathrm{HT}} > \hat{R}_{\mathrm{TPB}}$. Also as expected, we find that the runtime t_{grouper} (central panel of Fig. 9) of the HT Pauli grouping algorithm increases polynomially with the number of subgraphs s. In the bottom panel of Fig. 9, we display the break even number $N_{\mathrm{breakeven}}$ of energy estimations (i.e., function calls for estimating $\mathrm{Tr}[O\rho_i]$ for some quantum states $\rho_1, \dots, \rho_N$) after which the time saved on a superconducting quantum computer would outweigh the invested classical preprocessing time. Note that break even is reached much earlier for other quantum computing architectures or if the figure of merit is financial cost instead of time. We consider the same scenario as in SM Sec. VI, leading to a saved time per energy estimation given by

$$t_{\mathrm{saved/est.}} = 50\,\mu\mathrm{s} \times (1 - \hat{R}_{\mathrm{TPB}}/\hat{R}_{\mathrm{HT}}) \times 100M. \tag{47}$$

From this, we obtain

$$N_{\mathrm{breakeven}} = \lceil t_{\mathrm{grouper}}/t_{\mathrm{saved/est.}} \rceil . \tag{48}$$

We observe that break even can be reached after as little as 10^4 to 10^5 energy estimations. In practical scenarios, one typically requires many more energy estimations. Hence, before running a quantum simulation experiment with the Hamiltonians in Tab. XI, it would make sense to execute our Pauli grouping algorithm for an even larger number s to obtain even larger values of $\hat{R}_{\mathrm{HT}}$.

A complementary possibility to boost $\hat{R}_{\mathrm{HT}}$ is this: start with a HT Pauli grouping, e.g., the one for $n = 38$ and $s = 10^5$. Out of the $M = 53,214$ Pauli operators, there are $5,006$ which have been assigned to a size-1 group. By regrouping these $5,006$ Pauli operators with an increased number of subgraphs, $s = 10^6$, and an improved square-lattice connectivity, we can decrease the overall number of readout circuits from $13,626$ to $12,511$, which comes with an increase of $\hat{R}_{\mathrm{HT}}$ from 16.0815 to 16.1567. Such efforts can be worthwhile because of the reusability potential of Pauli groupings.

Finally, in Fig. 10, we use a reasonable hyperparameter choice to compute Pauli groupings for all ten Hamiltonians from Tab. XI. As expected, we observe that the performance of the HT Pauli grouper and the quality of the result as measured by $\hat{R}_{\mathrm{HT}}$ have only a little dependence on the interatomic distance d.

In conclusion, we have established that our method works reasonably well in the case of molecular Hamiltonians expressed in sophisticated basis sets. This is unsurprising as our method has been designed for general observables of the form $O = \sum_{i=1}^{M} c_i P_i$ with $M \in \mathcal{O}(\mathrm{poly}(n))$ to which these Hamiltonians belong.

[1] K. Temme, S. Bravyi, and J. M. Gambetta, Error mitigation for short-depth quantum circuits, Phys. Rev. Lett. **119**, 180509 (2017).

[2] S. Bravyi, S. Sheldon, A. Kandala, D. C. Mckay, and J. M. Gambetta, Mitigating measurement errors in multiqubit experiments, Phys. Rev. A **103**, 042605 (2021).

[3] P. D. Nation, H. Kang, N. Sundaresan, and J. M. Gambetta, Scalable mitigation of measurement errors on quantum computers, PRX Quantum **2**, 040326 (2021).

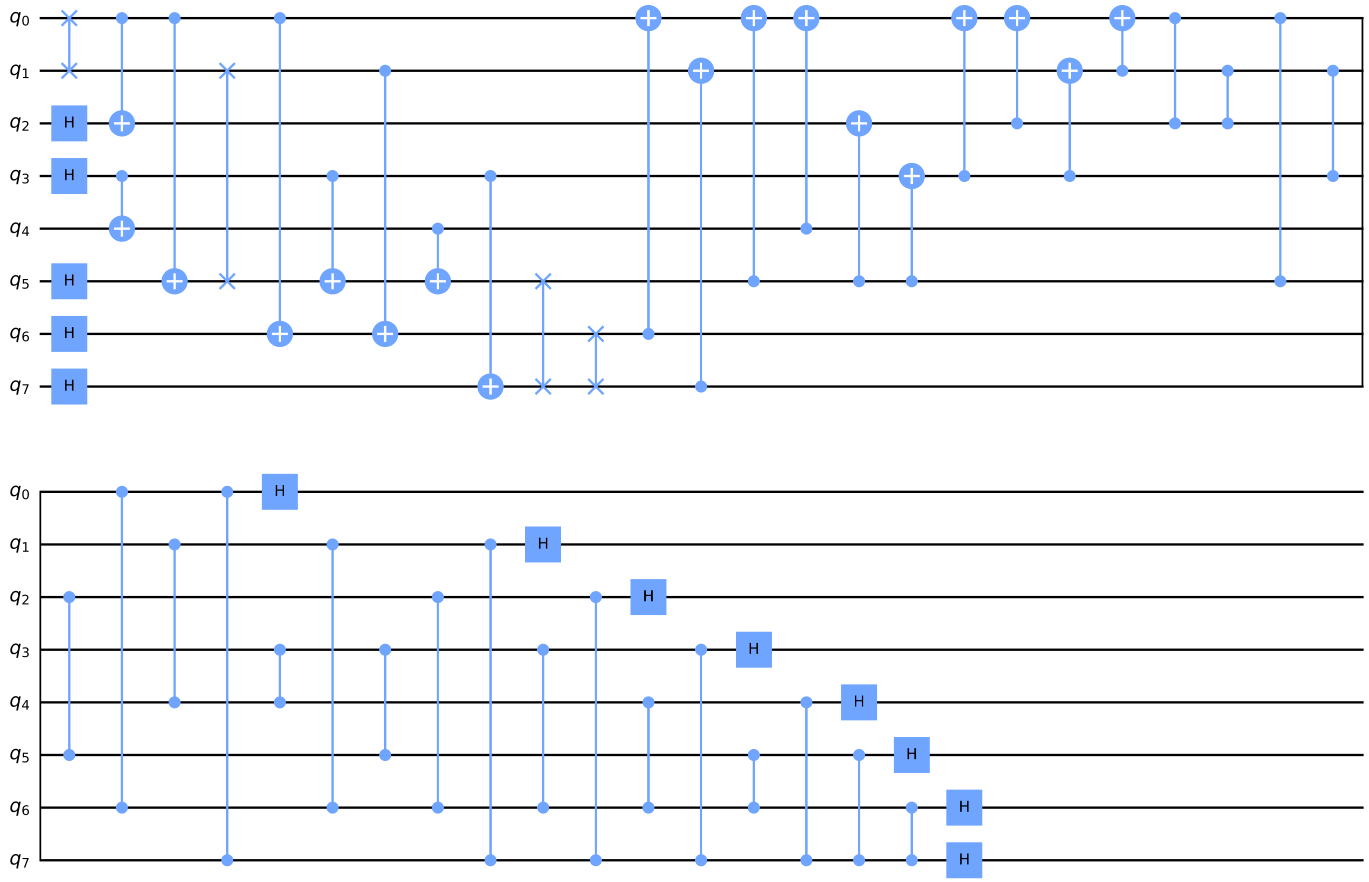

FIG. 8. GC readout circuit no. 3 from Tab. V, which diagonalizes IXXIIXXI, IYYZIXXI, IXXIIYYZ, IYYZIYYZ, ZIXXIIXX, IZYYZIXX, IZYYIZYY, ZIXXZZYY, IZYYIXXI, ZIXXZXXI, IZYYIYYZ, ZIXXZYYZ, IXXIZIXX, IYYZZIXX, IXXIIZYY, IYYZIZYY, IYZYIIII, ZYZYZIII, IIIIIYZY, and IIIIZYZY [5]. After being transpiled to a linear connectivity, this circuit contains 13 Hadamard, 18 Cnot, 21 Cphase, and 66 Swap gates. Note added: this circuit has been simplified in Fig. 3 of Ref. [40].

[4] O. Crawford, B. v. Straaten, D. Wang, T. Parks, E. Campbell, and S. Brierley, Efficient quantum measurement of Pauli operators in the presence of finite sampling error, Quantum **5**, 385 (2021).

[5] P. Gokhale, O. Angiuli, Y. Ding, K. Gui, T. Tomesh, M. Suchara, M. Martonosi, and F. T. Chong, $O(N^3)$ Measurement Cost for Variational Quantum Eigensolver on Molecular Hamiltonians, IEEE Trans. Quantum Eng. **1**, 1 (2020).

[6] D. A. Lidar and T. A. Brun, *Quantum Error Correction* (Cambridge University Press, 2013).

[7] J. Dehaene and B. De Moor, Clifford group, stabilizer states, and linear and quadratic operations over GF(2), Phys. Rev. A **68**, 042318 (2003).

[8] B. Wu, J. Sun, Q. Huang, and X. Yuan, Overlapped grouping measurement: A unified framework for measuring quantum states, Quantum **7**, 896 (2023).

[9] J. R. McClean, J. Romero, R. Babbush, and A. Aspuru-Guzik, The theory of variational hybrid quantum-classical algorithms, New J. Phys. **18**, 023023 (2016).

[10] W. J. Huggins, J. R. McClean, N. C. Rubin, Z. Jiang, N. Wiebe, K. B. Whaley, and R. Babbush, Efficient and noise resilient measurements for quantum chemistry on near-term quantum computers, npj Quantum Inf. **7** (2021).

[11] J. M. Kübler, A. Arrasmith, L. Cincio, and P. J. Coles, An adaptive optimizer for measurement-frugal variational algorithms, Quantum **4**, 263 (2020).

[12] R. Sweke, F. Wilde, J. Meyer, M. Schuld, P. K. Faehrmann, B. Meynard-Piganeau, and J. Eisert, Stochastic gradient descent for hybrid quantum-classical optimization, Quantum **4**, 314 (2020).

[13] A. Arrasmith, L. Cincio, R. D. Somma, and P. J. Coles, Operator sampling for shot-frugal optimization in variational algorithms, arXiv preprint arXiv:2004.06252 (2020).

[14] N. C. Rubin, R. Babbush, and J. McClean, Application of fermionic marginal constraints to hybrid quantum algorithms, New J. Phys. **20**, 053020 (2018).

[15] D. Wecker, M. B. Hastings, and M. Troyer, Progress towards practical quantum variational algorithms, Phys. Rev. A **92**, 042303 (2015).

[16] K. Phalak and S. Ghosh, Shot Optimization in Quantum Machine Learning Architectures to Accelerate Training, IEEE Access **11**, 41514 (2023).

[17] L. Zhu, S. Liang, C. Yang, and X. Li, Optimizing Shot Assignment in Variational Quantum Eigensolver Measurement, J. Chem. Theory Comput. **20**, 10.1021/acs.jctc.3c01113 (2024).

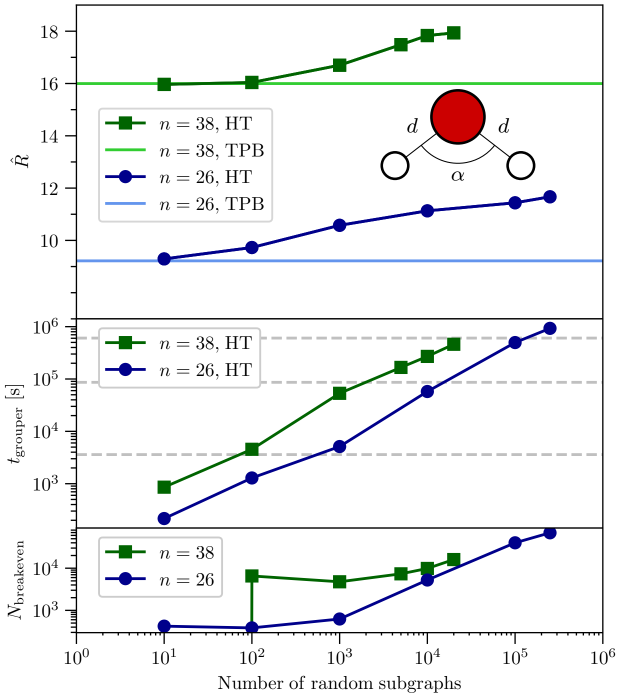

FIG. 9. Performance of Pauli Grouping Algorithm 1 for the water molecules from Tab. XI for a fixed OH distance of $d = 2.552\,\text{Å}$. We display the estimated shot reduction $\hat{R}$, the HT-grouper time t_{grouper}, and $N_{\text{breakeven}}$ from Eq. (48) as a function of the number of random subgraphs s (a hyperparameter in Algorithm 1). The readout circuits are tailored to a linear hardware connectivity. All computations were carried out on a 20 core Intel Xeon CPU E5-2697 v2 device.

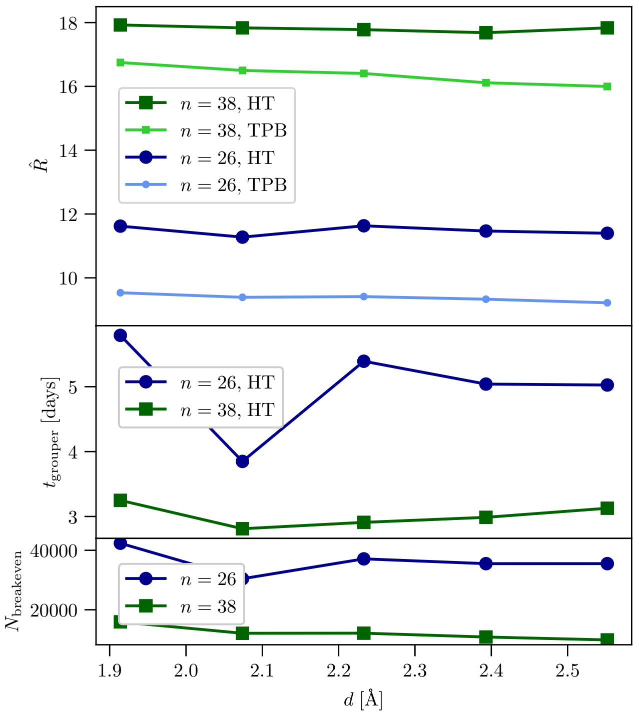

FIG. 10. Performance of Pauli Grouping Algorithm 1 for the water molecules from Tab. XI for a variable OH distance d. Here, the numbers of random subgraphs $s(n)$ are fixed to $s(26) = 10^5$ and $s(38) = 10^4$. The plotted quantities are the same as in Fig. 9. The readout circuits are tailored to a linear hardware connectivity. All computations were carried out on a 20 core Intel Xeon CPU E5-2697 v2 except for the data point at $n = 38$ and $d = 2.552\,\text{Å}$ which was executed on a 20 core Intel Xeon CPU E5-2680 v3 device and the data point at $n = 38$ and $d = 1.992\,\text{Å}$ which was executed on a 20 core AMD EPYC 7713.

[18] S. McArdle, S. Endo, A. Aspuru-Guzik, S. C. Benjamin, and X. Yuan, Quantum computational chemistry, Rev. Mod. Phys. **92**, 015003 (2020).

[19] V. Verteletskyi, T.-C. Yen, and A. F. Izmaylov, Measurement optimization in the variational quantum eigensolver using a minimum clique cover, J. Chem. Phys. **152** (2020).

[20] T.-C. Yen, V. Verteletskyi, and A. F. Izmaylov, Measuring All Compatible Operators in One Series of Single-Qubit Measurements Using Unitary Transformations, J. Chem. Theory Comput. **16**, 2400 (2020).

[21] A. Jena, S. N. Genin, and M. Mosca, Optimization of variational-quantum-eigensolver measurement by partitioning Pauli operators using multiqubit Clifford gates on noisy intermediate-scale quantum hardware, Phys. Rev. A **106**, 042443 (2022).

[22] S. B. Bravyi and A. Y. Kitaev, Fermionic Quantum Computation, Ann. Phys. (N. Y.) **298**, 210 (2002).

[23] A. Kandala, A. Mezzacapo, K. Temme, M. Takita, M. Brink, J. M. Chow, and J. M. Gambetta, Hardware-efficient variational quantum eigensolver for small molecules and quantum magnets, Nat. **549**, 242 (2017).

[24] `https://cloud.ibm.com/catalog/services/qiskit-runtime`.

[25] Q. Sun, T. C. Berkelbach, N. S. Blunt, G. H. Booth, S. Guo, Z. Li, J. Liu, J. D. McClain, E. R. Sayfutyarova, S. Sharma, S. Wouters, and G. K.-L. Chan, PySCF: the Python-based simulations of chemistry framework, Wiley Interd. Rev.: Comput. Mol. Sci. **8**, e1340 (2018).

[26] N. Moll, A. Fuhrer, P. Staar, and I. Tavernelli, Optimizing qubit resources for quantum chemistry simulations in second quantization on a quantum computer, J. Phys. A **49**, 295301 (2016).

[27] S. Bravyi, J. M. Gambetta, A. Mezzacapo, and K. Temme, Tapering off qubits to simulate fermionic Hamiltonians, arXiv:1701.08213 [quant-ph] (2017).

[28] K. Setia and J. D. Whitfield, Bravyi-Kitaev Superfast simulation of electronic structure on a quantum computer, J. Chem. Phys. **148**, 164104 (2018), 1712.00446.

[29] Qiskit: An Open-source Framework for Quantum Computing (2021).

[30] M. Miller and D. Miller, GraphStateVis: Interactive Visual Analysis of Qubit Graph States and their Stabilizer Groups, IEEE Trans. Quantum Eng. **1**, 378 (2021).

[31] A. Heckel, The chromatic number of dense random graphs, Random Struct. Algorithms **53**, 140 (2018).

[32] D. C. McKay, C. J. Wood, S. Sheldon, J. M. Chow, and J. M. Gambetta, Efficient Z gates for quantum

computing, Phys. Rev. A **96**, 022330 (2017).

[33] M. A. Nielsen and I. L. Chuang, *Quantum Computation and Quantum Information* (Cambridge University Press, 2000).

[34] H. F. Trotter, On the product of semi-groups of operators, Proc. Amer. Math. Soc. **10**, 545 (1959).

[35] D. Maslov, Linear depth stabilizer and quantum fourier transformation circuits with no auxiliary qubits in finite-neighbor quantum architectures, Phys. Rev. A **76**, 052310 (2007).

[36] C. H. Bennett, G. Brassard, C. Crépeau, R. Jozsa, A. Peres, and W. K. Wootters, Teleporting an unknown quantum state via dual classical and Einstein-Podolsky-Rosen channels, Phys. Rev. Lett. **70**, 1895 (1993).

[37] F. Huber and S. Severini, Some Ulam's reconstruction problems for quantum states, J. Phys. A Math. Theor. **51**, 435301 (2018).

[38] D. Miller, D. Loss, I. Tavernelli, H. Kampermann, D. Bruß, and N. Wyderka, Shor–Laflamme distributions of graph states and noise robustness of entanglement, J. Phys. A Math. Theor. **56**, 335303 (2023).

[39] R. Muller, Pyquante: Python quantum chemistry (accessed 25 February 2022).

[40] E. M. Murairi and M. J. Cervia, Reducing circuit depth with qubitwise diagonalization, Phys. Rev. A **108**, 062414 (2023).

[41] I. O. Sokolov, P. K. Barkoutsos, P. J. Ollitrault, D. Greenberg, J. Rice, M. Pistoia, and I. Tavernelli, Quantum orbital-optimized unitary coupled cluster methods in the strongly correlated regime: Can quantum algorithms outperform their classical equivalents?, J. Chem. Phys. **152**, 124107 (2020).

[42] J. R. McClean, N. C. Rubin, K. J. Sung, I. D. Kivlichan, X. Bonet-Monroig, Y. Cao, C. Dai, E. S. Fried, C. Gidney, B. Gimby, P. Gokhale, T. Häner, T. Hardikar, V. Havlíček, O. Higgott, C. Huang, J. Izaac, Z. Jiang, X. Liu, S. McArdle, M. Neeley, T. O'Brien, B. O'Gorman, I. Ozfidan, M. D. Radin, J. Romero, N. P. D. Sawaya, B. Senjean, K. Setia, S. Sim, D. S. Steiger, M. Steudtner, Q. Sun, W. Sun, D. Wang, F. Zhang, and R. Babbush, Openfermion: the electronic structure package for quantum computers, Quantum Science and Technology **5**, 034014 (2020).

TABLE VI. The Pauli operators P_i and their coefficients c_i (in Hartree) of the molecular Hamiltonian $O = \sum_{i=1}^{M} c_i P_i$ describing a four-site hydrogen chain with an interatomic distance of $d = 1.0\,\text{Å}$. Since the trivial Pauli operator $I^{\otimes 8}$ with coefficient $-0.33147\,\text{Ha}$ (which includes nuclear-nuclear repulsion) requires no measurement, we do not include it in the $M = 184$ term count. The relevant integrals in the STO-3G basis are obtained with Qiskit nature [29] in combination with pyquante [39]. Then, the Bravyi-Kitaev encoding is used to map the Hamiltonian to $n = 8$ qubits [22]. The operators are grouped into $N_{\text{TPB}} = 35$ tensor product bases (TPBs) using the SI-QWC algorithm (see SM Sec. V). Every TPB can be measured with a readout circuit that does not require any two-qubit gates; we follow the standard procedure of individually diagonalizing X and Y via H and $HS^\dagger$, respectively.

ZZZIZIII<br>−0.334612<br>IIIIZZZI<br>−0.334612<br>IIIZIIII<br>0.181365<br>IIIIIIIZ<br>0.181365<br>ZZZIIZZI<br>0.145262<br>IIIZZZZI<br>0.130739<br>ZZZIZIIZ<br>0.130739<br>IIIZIIIZ<br>0.124322<br>ZZZIZZII<br>0.120054<br>IZIIZZZI<br>0.120054<br>IZIIIZII<br>0.116851<br>IIZZZZZI<br>0.115986<br>ZZZIZIZZ<br>0.115986<br>IIZZIIZZ<br>0.113407<br>IIZZIZII<br>0.111941<br>IZIIIIZZ<br>0.111941<br>IIIZIZII<br>0.111498<br>IZIIIIIZ<br>0.111498<br>IIZZIIIZ<br>0.108983<br>IIIZIIZZ<br>0.108983<br>ZZZZZIII<br>0.106471<br>IIIIZZZZ<br>0.106471<br>ZZIZZIII<br>0.089825<br>IIIIZZIZ<br>0.089825<br>IIIIIIZZ<br>0.087927<br>IIZZIIII<br>0.087927<br>IZIZIIII<br>0.084540<br>IIIIIZIZ<br>0.084540<br>ZIZIZIII<br>0.079439<br>IIIIZIZI<br>0.079439<br>IIIIIZII<br>−0.079044<br>IZIIIIII<br>−0.079044<br>IZZZIIII<br>0.077620<br>IIIIIZZZ<br>0.077620<br>IIZIIIII<br>0.069638<br>IIIIIIZI<br>0.069638	IXIIIXII<br>0.040616<br>ZXZIZXII<br>−0.040616<br>ZXZIIXZI<br>0.040616<br>IXIIZXZI<br>−0.040616<br>IIIXIIIX<br>0.039345<br>IIZXIIIX<br>−0.039345<br>IIIXIIZX<br>−0.039345<br>IIZXIIZX<br>0.039345<br>IIIXIXII<br>−0.037658<br>IIZXIXII<br>0.037658<br>IIIXZXZI<br>0.037658<br>IIZXZXZI<br>−0.037658<br>IXIIIIIX<br>−0.037658<br>ZXZIZIIX<br>0.037658<br>IXIIIIZX<br>0.037658<br>ZXZIZIZX<br>−0.037658<br>IXIXIIII<br>−0.024418<br>ZXIXZIII<br>−0.024418<br>IIIIIXIX<br>−0.024418<br>IIIIZXIX<br>−0.024418<br>IXZXIIII<br>0.013030<br>ZXZXZIII<br>0.013030<br>IIIIIXZX<br>0.013030<br>IIIIZXZX<br>0.013030	IZXIIZXI<br>0.026161<br>ZIXZZZXI<br>−0.026161<br>IZXIZIXZ<br>−0.026161<br>ZZXZZIII<br>−0.025545<br>IIIIZZXZ<br>−0.025545<br>IZXIIIIZ<br>0.021062<br>IIIZIZXI<br>0.021062<br>IIIIIZXI<br>−0.017265<br>IZXIIIII<br>−0.017265<br>IZXZIIII<br>0.010291<br>IIIIIZXZ<br>0.010291<br>IZXIIZII<br>0.000703<br>ZIXZZZII<br>−0.000703<br>IZIIIZXI<br>0.000703<br>IZIIZIXZ<br>−0.000703	IXXIIXXI<br>0.034321<br>ZIXZIIXZ<br>0.026161<br>IIXIIIII<br>0.025545<br>IIIIIIXI<br>0.025545<br>IXXIIXII<br>0.024842<br>IXIIIXXI<br>0.024842<br>ZIXZZIIZ<br>−0.021062<br>IIIZZIXZ<br>−0.021062<br>IIIIZIXZ<br>0.017265<br>ZIXZZIII<br>0.017265<br>ZIXIZIII<br>−0.010291<br>IIIIZIXI<br>−0.010291	ZXZIZXXI<br>−0.024842<br>IIIXIXXI<br>−0.024501<br>IIZXIXXI<br>0.024501<br>IIIXZIXX<br>0.010771<br>IIZXZIXX<br>−0.010771<br>ZXZIIIXX<br>0.010243<br>IXIIZIXX<br>−0.010243	IXXIZXZI<br>−0.024842<br>IXXIIIIX<br>−0.024501<br>IXXIIIZX<br>0.024501<br>ZIXXZIIX<br>0.010771<br>ZIXXZIZX<br>−0.010771<br>ZIXXZXII<br>−0.010243<br>$ZIXXIXZI$<br>0.010243	IXXIIYYZ<br>0.034321<br>IXIIIYYZ<br>0.024842<br>IIIXIYYZ<br>−0.024501<br>IXXXIIII<br>−0.022049<br>ZIXXZYYZ<br>0.013240<br>ZXXXZIII<br>0.011234	IYYZIXXI<br>0.034321<br>IYYZIXII<br>0.024842<br>IYYZIIIX<br>−0.024501<br>IIIIIXXX<br>−0.022049<br>IYYZZIXX<br>0.013240<br>IIIIZXXX<br>0.011234	IYZYIIII<br>−0.011388<br>ZYZYZIII<br>−0.011388<br>IIIIIYZY<br>−0.011388<br>IIIIZYZY<br>−0.011388
IYXYIIII<br>−0.022049<br>IIIIIYXY<br>−0.022049<br>ZYXYZIII<br>0.011234<br>IIIIZYXY<br>0.011234	ZZZIZZXI<br>0.023385<br>ZZZIIIXZ<br>−0.023385<br>IIZZIZXI<br>0.001014<br>IIZZZIXZ<br>−0.001014	ZIXZIZZI<br>−0.023385<br>IZXIZZZI<br>0.023385<br>IZXIIIZZ<br>0.001014<br>ZIXZZIZZ<br>−0.001014	ZIXXZZYY<br>0.024268<br>IXXIIZYY<br>0.013240<br>IIIXIZYY<br>0.010771<br>IXIIIZYY<br>−0.010243	IZYYZIXX<br>0.024268<br>IZYYIXXI<br>0.013240<br>IZYYIIIX<br>0.010771<br>IZYYIXII<br>−0.010243	IZXIIXYY<br>0.024628<br>ZIXZZXYY<br>−0.024628<br>IIIZIXYY<br>0.020392<br>IZIIIXYY<br>0.001715	IZXIIYYX<br>−0.024628<br>ZIXZZYYX<br>0.024628<br>IIIZIYYX<br>−0.020392<br>IZIIIYYX<br>−0.001715	IXYYIZXI<br>0.024628<br>IXYYZIXZ<br>−0.024628<br>IXYYIIIZ<br>0.020392<br>IXYYIZII<br>0.001715	IYYXIZXI<br>−0.024628<br>IYYXZIXZ<br>0.024628<br>IYYXIIIZ<br>−0.020392<br>IYYXIZII<br>−0.001715
ZIXXIIXX<br>0.024268<br>ZIXXZXXI<br>0.013240<br>IXXIZIXX<br>0.013240	IYYXIYYX<br>0.026958<br>IIIIIYYX<br>−0.005561<br>IYYXIIII<br>−0.005561	IIZXIZYY<br>−0.010771<br>ZXZIZZYY<br>0.010243	IZYYIIZX<br>−0.010771<br>IZYYZXZI<br>0.010243	ZZZIZXYY<br>0.021477<br>IIZZIXYY<br>−0.002452	ZZZIZYYX<br>−0.021477<br>IIZZIYYX<br>0.002452	IXYYZZZI<br>0.021477<br>IXYYIIZZ<br>−0.002452	IYYXZZZI<br>−0.021477<br>IYYXIIZZ<br>0.002452	ZXZIZYYZ<br>−0.024842<br>IIZXIYYZ<br>0.024501
IYYZZXZI<br>−0.024842<br>IYYZIIZX<br>0.024501	IYYXIXYY<br>−0.026958<br>IIIIIXYY<br>0.005561	IXYYIYYX<br>−0.026958<br>IXYYIIII<br>0.005561	IYYZIZYY<br>0.013240	IZYYIYYZ<br>0.013240	IZYYIZYY<br>0.024268	IXYYIXYY<br>0.026958	IYYZIYYZ<br>0.034321	

TABLE VII. Nine of the $N_{\mathrm{HT}} = 10$ hardware-tailored (HT) diagonalization circuits for the H_4-Hamiltonian from Tab. VI. For visual reasons, we do not depict the trivial circuit which "diagonalizes" the TPB $Z^{\otimes 8}$. The circuits are tailored to a linear connectivity. Note that only 4 different circuit templates are selected by Algorithm 1, even though we considered the exhaustive list of all 128 subgraphs of the 8-vertex path graph. Thus, it might be possible to analytically construct HT diagonalization circuits for hydrogen chains with $n \gg 8$ qubits, see Tab. II in the main text for a starting point.

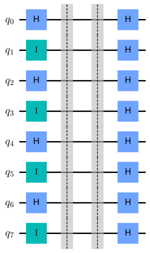

Non-diagonal	Diagonalized
IXIIZXZI	+IZIIZZZI
ZXZIIXZI	+ZZZIIZZI
ZXZIZXII	+ZZZIZZII
IXIIIXII	+IZIIIZII
IIZXIIZX	+IIZZIIZZ
IIIXIIZX	+IIIZIIZZ
IIZXIIIX	+IIZZIIIZ
IIIXIIIX	+IIIZIIIZ
ZXZIZIZX	+ZZZIZIZZ
IXIIIIZX	+IZIIIIZZ
ZXZIZIIX	+ZZZIZIIZ
IXIIIIIX	+IZIIIIIZ
IIZXZXZI	+IIZZZZZI
IIIXZXZI	+IIIZZZZI
IIZXIXII	+IIZZIZII
IIIXIXII	+IIIZIZII
IIIIZXIX	+IIIIZZIZ
IIIIIXIX	+IIIIIZIZ
ZXIXZIII	+ZZIZZIII
IXIXIIII	+IZIZIIII
IIIIZXZX	+IIIIZZZZ
IIIIIXZX	+IIIIIZZZ
ZXZXZIII	+ZZZZZIII
IXZXIIII	+IZZZIIII

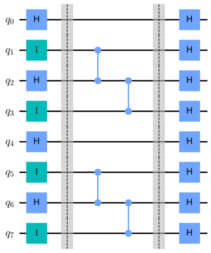

Non-diagonal	Diagonalized
IYYZIYYZ	+IZZIIZZI
IXXIIYYZ	-IZIIIZZI
IYYZIXXI	-IZZIIZII
IXXIIXXI	+IZIIIZII
ZIXXZZYY	-ZIIZZIZZ
IZYYIZYY	+IIZZIIZZ
IZYYZIXX	-IIZZZIIZ
ZIXXIIXX	+ZIIZIIIZ
IYYZIZYY	+IZZIIIZZ
IXXIIZYY	-IZIIIIZZ
IYYZZIXX	-IZZIZIIZ
IXXIZIXX	+IZIIZIIZ
ZIXXZYYZ	-ZIIZZZZI
IZYYIYYZ	+IIZZIZZI
ZIXXZXXI	+ZIIZZZII
IZYYIXXI	-IIZZIZII
IIIIZYZY	-IIIIZZZZ
IIIIIYZY	-IIIIIZZZ
ZYZYZIII	-ZZZZZIII
IYZYIIII	-IZZZIIII

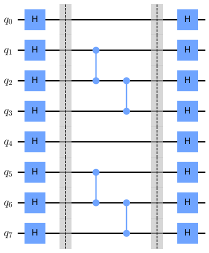

Non-diagonal	Diagonalized
IXYYIXYY	+IIZZIIZZ
IYYXIXYY	+IZZIIIZZ
IXYYIYYX	+IIZZIZZI
IYYXIYYX	+IZZIIZZI
IZXIZIXZ	+IZIIZIIZ
ZIXZIIXZ	+ZIIZIIIZ
ZIXZZZXI	+ZIIZZZII
IZXIIZXI	+IZIIIZII
ZIXZZXYY	+ZIIZZIZZ
IZXIIXYY	+IZIIIIZZ
ZIXZZYYX	+ZIIZZZZI
IZXIIYYX	+IZIIIZZI
IXYYZIXZ	+IIZZZIIZ
IYYXZIXZ	+IZZIZIIZ
IXYYIZXI	+IIZZIZII
IYYXIZXI	+IZZIIZII
ZIXZZIII	+ZIIZZIII
IZXIIIII	+IZIIIIII
IIIIZIXZ	+IIIIZIIZ
IIIIIZXI	+IIIIIZII
IXYYIIII	+IIZZIIII
IYYXIIII	+IZZIIIII
IIIIIXYY	+IIIIIIZZ
IIIIIYYX	+IIIIIZZI

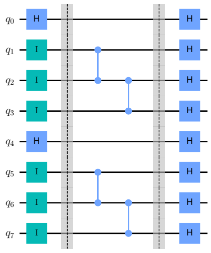

Non-diagonal	Diagonalized
IIIIZZXZ	+IIIIZIZI
ZZXZZIII	+ZIZIZIII
ZXZIZYYZ	+ZZIIZZZI
IYYZZXZI	+IZZIZZII
IIZXIYYZ	+IIIZIZZI
IYYZIIZX	+IZZIIIIZ
IIIIIYXY	-IIIIIZZZ
IYXYIIII	-IZZZIIII
IIIIZYXY	-IIIIZZZZ
ZYXYZIII	-ZZZZZIII
IIZXIZYY	+IIIZIIZZ
IZYYIIZX	+IIZZIIIZ
IIIIIZXZ	+IIIIIIZI
IZXZIIII	+IIZIIIII
ZXZIZZYY	+ZZIIZIZZ
IZYYZXZI	+IIZZZZII

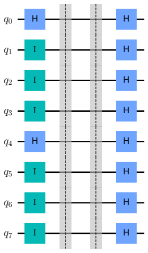

Non-diagonal	Diagonalized
IIIIIIXI	+IIIIIIZI
IIXIIIII	+IIZIIIII
IXIIIXXI	+IZIIIZZI
IXXIIXII	+IZZIIZII
IIIXIXXI	+IIIZIZZI
IXXIIIIX	+IZZIIIIZ
IIIIIXXX	+IIIIIZZZ
IXXXIIII	+IZZZIIII
IIIIZXXX	+IIIIZZZZ
ZXXXZIII	+ZZZZZIII
IIIXZIXX	+IIIZZIZZ
ZIXXZIIX	+ZIZZZIIZ
IIIIZIXI	+IIIIZIZI
ZIXIZIII	+ZIZIZIII
IXIIZIXX	+IZIIZIZZ
ZIXXZXII	+ZIZZZZII

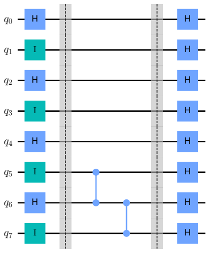

Non-diagonal	Diagonalized
IXIIIYYZ	-IZIIIZZI
ZXZIZXXI	+ZZZIZZII
IIIXIYYZ	-IIIZIZZI
IIZXIXXI	+IIZZIZII
IIIXIZYY	-IIIZIIZZ
IIZXZIXX	+IIZZZIIZ
IXIIIZYY	-IZIIIIZZ
ZXZIIIXX	+ZZZIIIIZ

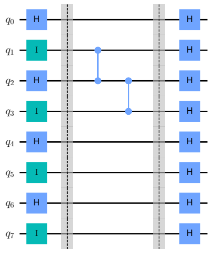

Non-diagonal	Diagonalized
IXXIZXZI	+IZIIZZZI
IYYZIXII	-IZZIIZII
IXXIIIZX	+IZIIIIZZ
IYYZIIIX	-IZZIIIIZ
ZIXXZIZX	+ZIIZZIZZ
IZYYIIIX	-IIZZIIIZ
ZIXXIXZI	+ZIIZIZZI
IZYYIXII	-IIZZIZII

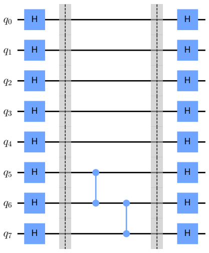

Non-diagonal	Diagonalized
ZZZIIIXZ	+ZZZIIIIZ
ZZZIZZXI	+ZZZIZZII
ZZZIZXYY	+ZZZIZIZZ
ZZZIZYYX	+ZZZIZZZI
IIIZZIXZ	+IIIZZIIZ
IIIZIZXI	+IIIZIZII
IIIZIXYY	+IIIZIIZZ
IIIZIYYX	+IIIZIZZI
IIZZIXYY	+IIZZIIZZ
IIZZIYYX	+IIZZIZZI
IZIIIXYY	+IZIIIIZZ
IZIIIYYX	+IZIIIZZI
IIZZZIXZ	+IIZZZIIZ
IIZZIZXI	+IIZZIZII
IZIIZIXZ	+IZIIZIIZ
IZIIIZXI	+IZIIIZII

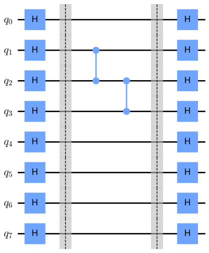

Non-diagonal	Diagonalized
IZXIZZZI	+IZIIZZZI
ZIXZIZZI	+ZIIZIZZI
IXYYZZZI	+IIZZZZZI
IYYXZZZI	+IZZIZZZI
ZIXZZIIZ	+ZIIZZIIZ
IZXIIIIZ	+IZIIIIIZ
IXYYIIIZ	+IIZZIIIZ
IYYXIIIZ	+IZZIIIIZ
IXYYIIZZ	+IIZZIIZZ
IYYXIIZZ	+IZZIIIZZ
IXYYIZII	+IIZZIZII
IYYXIZII	+IZZIIZII
ZIXZZIZZ	+ZIIZZIZZ
IZXIIIZZ	+IZIIIIZZ
ZIXZZZII	+ZIIZZZII
IZXIIZII	+IZIIIZII

TABLE VIII. Random choice of parameters $\theta_j, \phi_j, \lambda_j \in [0, 2\pi]$. This defines the state $|\Psi\rangle = \bigotimes_{j=1}^{8} U_3(\theta_j, \phi_j, \lambda_j)|0\rangle$ that is prepared and measured in our experiment.

j	1	2	3	4	5	6	7	8
θ_j	1.203	4.935	1.737	5.504	4.294	3.526	4.856	3.867
ϕ_j	3.909	4.901	5.038	2.248	4.478	3.161	5.546	0.474
λ_j	2.750	1.713	6.020	3.148	2.326	0.087	2.293	2.317

TABLE IX. Optimal shot-allocation ratios for Pauli groupings of the Hamiltonian $O = \sum_{i=1}^{184} c_i P_i$ into TPBs (Tab. VI), GC (Tab. V), and HT collections (Tab. VII). The variances $\mathrm{Var}[O_i] = \langle\Psi| O_i^2 |\Psi\rangle - \langle\Psi| O_i |\Psi\rangle^2$ that enter Eq. (10) in SM Sec. IV are computed for the target state $|\Psi\rangle$ defined in Tab. VIII.

Collection	TPB	GC	HT
1	0.38529	0.53671	0.52623
2	0.08011	0.11160	0.10942
3	0.03069	0.06248	0.06126
4	0.01975	0.05711	0.05600
5	0.03104	0.04949	0.05040
6	0.01688	0.05166	0.04766
7	0.01478	0.05390	0.03751
8	0.01355	0.03727	0.03620
9	0.01455	0.03977	0.03676
10	0.01326		0.03856
11	0.03121		
12	0.02191		
13	0.01637		
14	0.02381		
15	0.01706		
16	0.01653		
17	0.01656		
18	0.02084		
19	0.02083		
20	0.01561		
21	0.01918		
22	0.01194		
23	0.01468		
24	0.01624		
25	0.01840		
26	0.01894		
27	0.01096		
28	0.01097		
29	0.01066		
30	0.01066		
31	0.00651		
32	0.00647		
33	0.00953		
34	0.00698		
35	0.00727		

TABLE X. Device specification of the 127-qubit quantum processor *ibm_washington*. The experiment was carried out on a linear chain of eight qubits (42, 43, 44, 45, 54, 64, 63, and 62). For every qubit, we provide the relaxation time T_1, coherence time T_2, error rates of the $\sqrt{X}$ gate, and the readout error probability $P(m\,|\,p)$ for preparing $|p\rangle$ but measuring $|m\rangle$. Note that every single-qubit gate is a sequence of $\sqrt{X}$ gates and virtual z rotations, which have a duration of 36 ns and 0 ns, respectively [32]. Readout pulses have a duration of 864 ns. Finally, we provide the error rate and duration of a CNOT gate with control qubit i and target qubit j, where $(i, j) \in \{(42, 43), (43, 44), (44, 45), (45, 54), (54, 64), (64, 63), (63, 62)\}$.

Qubit	T_1 [µs]	T_2 [µs]	$P(0\,|\,1)$	$P(1\,|\,0)$	$\sqrt{X}$ gate error	CNOT gate error	CNOT gate duration [ns]
42	108.62	255.06	0.012	0.009	0.000139	0.014673	804
43	128.82	195.74	0.025	0.025	0.000299	0.012446	427
44	89.34	123.60	0.019	0.015	0.000299	0.014925	1074
45	107.16	158.24	0.042	0.025	0.000215	0.033060	597
54	111.27	117.25	0.015	0.010	0.000265	0.009645	377
64	99.75	90.05	0.011	0.012	0.000345	0.014083	405
63	83.30	174.79	0.013	0.004	0.000663	0.011548	548
62	81.93	142.67	0.052	0.007	0.000180		

TABLE XI. Water Hamiltonians expressed in complex molecular basis sets. The relevant integrals are obtained with OpenFermion [42] in combination with pyquante [39]. Applying the Bravyi-Kitaev mapper results in an n-qubit Hamiltonian $O = \sum_{i=1}^{M} c_i P_i$ with $c_i \in \mathbb{R}$ and Pauli operators $P_i \in \{I, X, Y, Z\}^{\otimes n}$. Note that the identity operator $P_0 = I^{\otimes n}$ is excluded from the term count M.

Basis Set	n	d	M
6-31G	26	1.914 Å	13,148
		2.074 Å	13,068
		2.233 Å	13,160
		2.393 Å	13,166
		2.552 Å	12,792
6-31G*	38	1.914 Å	53,692
		2.074 Å	53,698
		2.233 Å	53,666
		2.393 Å	53,712
		2.552 Å	53,214
